# A systematic review of home-based records in maternal and child health for improving informational continuity, health outcomes, and perceived usefulness in low and middle-income countries

**DOI:** 10.1371/journal.pone.0267192

**Published:** 2022-08-04

**Authors:** Linju Joseph, Anna Lavis, Sheila Greenfield, Dona Boban, Prinu Jose, Panniyammakal Jeemon, Semira Manaseki-Holland

**Affiliations:** 1 Institute of Applied Health Research, College of Medical and Dental Sciences, University of Birmingham, Edgbaston, Birmingham, United Kingdom; 2 Amrita Institute of Medical Sciences and Research Centre, Cochin, India; 3 Public Health Foundation of India, New Delhi, India; 4 Sree Chitra Tirunal Institute for Medical Sciences and Technology, Trivandrum, Kerala, India; Njala University, SIERRA LEONE

## Abstract

**Background:**

Evidence shows that a gap in the documentation of patients’ past medical history leads to errors in, or duplication of, treatment and is a threat to patient safety. Home-based or patient-held records (HBR) are widely used in low and middle-income countries (LMIC) in maternal and childcare. The aim is to systematically review the evidence on HBRs in LMICs for (1) improving informational continuity for providers and women/families across health care visits and facilities, (2) to describe the perceived usefulness by women/families and healthcare providers, and (3) maternal and child health outcomes of using HBRs for maternal and child health care.

**Methods:**

The protocol was registered in PROSPERO (CRD42019139365). We searched MEDLINE, EMBASE, CINAHL, and Global Index Medicus databases for studies with home-based records from LMICs. Search terms pertained to women or parent-held records and LMICs. Two reviewers assessed studies for inclusion using a priori study selection criteria- studies explaining the use of HBRs in LMIC for maternal and child health care. The included study quality was appraised using the Mixed Methods Appraisal Tool (MMAT). Results from all study designs were summarised narratively.

**Results:**

In total, 41 papers were included in the review from 4514 potential studies. Included studies represented various study designs and 16 countries. The least evaluated function of HBR was information continuity across health care facilities (n = 6). Overall, there were limited data on the usefulness of HBRs to providers and mothers/families. Home-based records were mostly available for providers during health care visits. However, the documentation in HBRs varied. The use of HBRs is likely to lead to improved antenatal visits and immunisation uptake, and skilled birth delivery in some settings. Mothers’ knowledge of breastfeeding practices and danger signs in pregnancy improved with the use of HBRs. One randomised trial found the use of HBRs reduced the risk of cognitive development delay in children and another reported on trial lessened the risk of underweight and stunted growth in children.

**Conclusion:**

There is limited literature from LMICs on the usefulness of HBRs and for improving information transfer across healthcare facilities, or their use by women at home. Current HBRs from LMICs are sub-optimally documented leading to poor informational availability that defeats the point of them as a source of information for future providers.

## Introduction

Quality of care is systematically deficient in most low and middle-income countries (LMIC) such that mothers and children receive less than half of the recommended clinical care during antenatal to post-natal and paediatric visits [1]. The World Health Organisation (WHO) has recognised the need for better information systems and documentation to improve outcomes for mothers and children [2]. Delays or errors in decision-making processes due to an inadequate maternity history or case documentation can contribute to complications in pregnancy, especially high-risk pregnancies [3, 4]. Pregnancy and childbirth involve several visits to healthcare providers (HCP) for ante-natal care, at the time of delivery, for post-partum care and vaccinations for the new-born [5]. These may involve different HCPs such as community healthcare workers, primary care nurses, general physicians, and obstetricians, and different health care facilities such as clinics or hospitals [6, 7]. When clinical handover takes place at these transition points, adequate medical information exchange regarding patients between HCPs and patients/families is necessary for ensuring continuity of care and patient safety [8, 9].

A home-based record (HBR) is a patient-held health document used to record the history of health services received by women/children, widely used in LMICs [10]. It can take various forms such as ante-natal records/cards, vaccination cards, or maternal and child handbooks and requires mothers to take the record to each visit to healthcare providers (HCP). In 2018, the WHO recommended the use of an HBR by all pregnant women to improve continuity and quality of care throughout pregnancy to child health care [11]. The primary function of an HBR in this context is to record essential information related to maternal, new-born, and child health (MNCH), including health status, visits to a health care provider, vaccinations received, and the child’s growth and development. Additionally, an HBR is intended to be used at home by mothers/caregivers for managing care (looking for danger signs, as a reminder for children’s vaccinations, etc.). One of the advantages of having a HBR is that all HCPs will write in the same record, which can help to reduce clinical errors and improve communication between HCPs, especially across different facilities [12]. This documented information is intended to equip all HCPs, especially frontline healthcare workers, with a standardised patient history, to make informed decisions on care and immunisation services [10].

Although HBRs has been used in many LMICs [10, 13], the evidence for whether they improve informational continuity for clinicians in those settings has not been reviewed rigorously. Previous systematic reviews [5, 12, 14, 15] have found that mothers and children with records tend to have better clinical outcomes such as improved antenatal care and reduced likelihood of pregnancy complications, and they have shown an increase in mother and child vaccination rates. Another review evaluating the effectiveness of women carrying case notes found no studies reporting the availability of records at the time of delivery [12]. Currently, there is insufficient evidence on the availability of documented clinical information for HCPs (across visits and healthcare facilities) using HBRs in LMICs. Additionally, there is little information on how HBRs are being used in routine practice in LMICs [16]. Given that, HBRs are widely implemented, recommended by WHO and often the only available medical records in LMICs, we aimed to systematically review the evidence of HBRs for women/families in LMICs. Specifically, for improving the informational continuity for HCPs across visits and healthcare facilities and communicating with HCPs and women/families. The findings can further aid in designing appropriate HBRs that can contribute to optimizing implementation of HBRs in LMICs. Further, we summarised the evidence of use and health outcomes of using HBRs by HCPs and women/families.

## Methods

The protocol for this review is registered with the PROSPERO International Prospective Register of Systematic Reviews (CRD42019139365) [17].

### Eligibility of studies

Each study had to meet the criteria set out in Table 1 to be included in the review. Studies needed to be done in LMICs as per the World Bank 2018 [18], have an HBR intervention for pregnant women/children such as antenatal records/cards, vaccination cards or maternal and child handbooks (MCH) and at least one measure relating to an outcome of HBR intervention such as availability of HBRs at consultations, improvements in antenatal visits/vaccination rates, etc. published in the English language.

**Table 1:** Inclusion and exclusion criteria for studies.

Characteristics	Inclusion	Exclusion
Population	Women/parents in LMIC, HCPs in LMICs	Women/parents and HCPs living in HIC
Intervention	Paper-based patient-held record (PHR), also known as home-based records. It can take various forms such as antenatal records/cards, vaccination cards, or maternal and child handbooks (MCH) and requires women/mothers to carry these records to each visit to healthcare providers (HCP).	Facility-based medical records, non-maternal patient-held records
Outcomes	(a) Informational continuity	Studies which do not provide details on information availability to HCPs/patients and patients/HCPs perceived view of information available in HBR.
	The availability of patient medical information for HCPs forms the basis of informational continuity. It involves patients carrying records to healthcare visits and HCPs documenting in the records. For this review, the information available in HBRs available at visits for HCPs at antenatal visits, at the time of hospital admission for maternal/childcare, at post-natal healthcare visits, and childcare visits such as vaccination history. It can be presented as frequencies or number of patients carrying the records to visits or as the prevalence of written clinical information availability for HCPs at visits. Views of patients carrying/not carrying HBRs. HCP views on the availability of HBRs for them to make clinical decisions, record the healthcare services and challenges in using HBRs	Studies that report only the distribution and coverage of HBRs.
	Data on quality of information recorded and available such as completeness of the records, the accuracy of the information, and clarity or legibility of handwritten information.	
	(b) Perceived usefulness of HBR	
	For the review, usefulness is defined as perceptions of women/family members/HCPs using HBRs, satisfaction with use, usability in terms of ease of reading or recording in the records, and or degree to which a HCP believes that using HBR improves their job, the function PHRs serve for HCPs, and women/families.	
	Health outcomes following the use of HBRs.	
	(c) Maternal, new-born, and child health outcomes as per WHO guidelines such as a change in maternal/neo-natal mortality, behavioural outcomes such as improvement in antenatal visits, improvement in vaccination rates, knowledge, attitude and practice changes	

### Search strategy

We systematically searched the literature using the electronic databases MEDLINE, EMBASE, and CINAHL from database inception to September 2020. Additional database search was done in GIM (Global Index Medicus) from database inception to August 2021 to include studies from LMIC. The reference lists and citations of full-text articles were hand-searched for additional eligible references. The search strategy was developed using search terms about home-based records and country names from the World Bank 2018 [18], by income classification. Search terms included (women-held OR parent-held OR mother OR pregnant women OR caregiver) AND (record OR handbook OR card OR notes) AND a list of LMICs. The search strategy developed for MEDLINE is available in the online supplement (S1 Box in S1 File). Further, we carried out additional searches on the websites of the WHO and the Japan International Co-operation Agency (JICA).

### Data extraction

Two reviewers (LJ and BD) extracted data from studies that met eligibility. LJ reviewed all extracted data. The data extracted were the study objective, population, and type of PHR, outcomes assessed, and results. Any disagreements in the data extraction were finalised after discussion with a third reviewer (SMH).

### Quality appraisal

For assessing the quality of included studies, the Mixed Methods Appraisal Tool (MMAT) version 2018 [19] was used. We assessed the quality of included studies using, ‘high’, ‘medium’ and ‘low’ descriptors. The studies that met all five criteria for quantitative and qualitative studies based on MMAT, were termed high, while studies that met three or four criteria were deemed to be medium, and finally, studies which met one or two criteria were classified as low. For mixed methods studies, the lowest score of the quantitative or qualitative strand was taken as the overall quality of the study.

### Data synthesis

Due to the wide variability of included study designs and outcomes, a meta-analysis was not conducted and the study findings were summarized narratively. The functions of HBRs in the included studies were summarized by using functions proposed by Osaki et al. [20] and Brown et al. [16] (Online supplement; S1 Table in S1 File) with elements that define handover communication. Therefore, functions of HBR were classified as

- **A Handover communication and information tool for HCPs**.- as a tool for recording health care received by women/children and monitoring by HCPs during clinic visits (primary provider) and across different health care facilities (for referral and transfer of information across HCPs/healthcare facilities).
- **A Handover communication and information tool from HCPs to women/families**-as a tool for receiving own documented medical information of the care received from HCPs, communication on further follow-up, and own care at home.

There are different records such as maternal cards, vaccination cards, maternal and child health handbooks, etc. (Online supplement; S1 Box in S1 File) included in the review and will be referred to as HBRs.

#### Outcomes

The outcome variables for informational continuity were summarised as availability or patients carrying HBRs to healthcare facilities, patient perception about carrying HBRs, completeness of the HBRs, and views of HCPs about the role of HBRs in improving information availability. The perceived usefulness of HBR variables was patients’ and HCPs’ satisfaction in using HBR, ease of recording or reading, patients’ and HCPs’ on how it was important for them, etc. Additionally, outcomes were considered relating to HBR as a handover communication tool between HCPs and mothers/children, such as, maternal and child health outcomes, health service utilisation, improvement in maternal and childcare behaviours such as breastfeeding. Both quantitative and qualitative findings were synthesised under each outcome.

## Results

### Study selection

**Fig 1:**
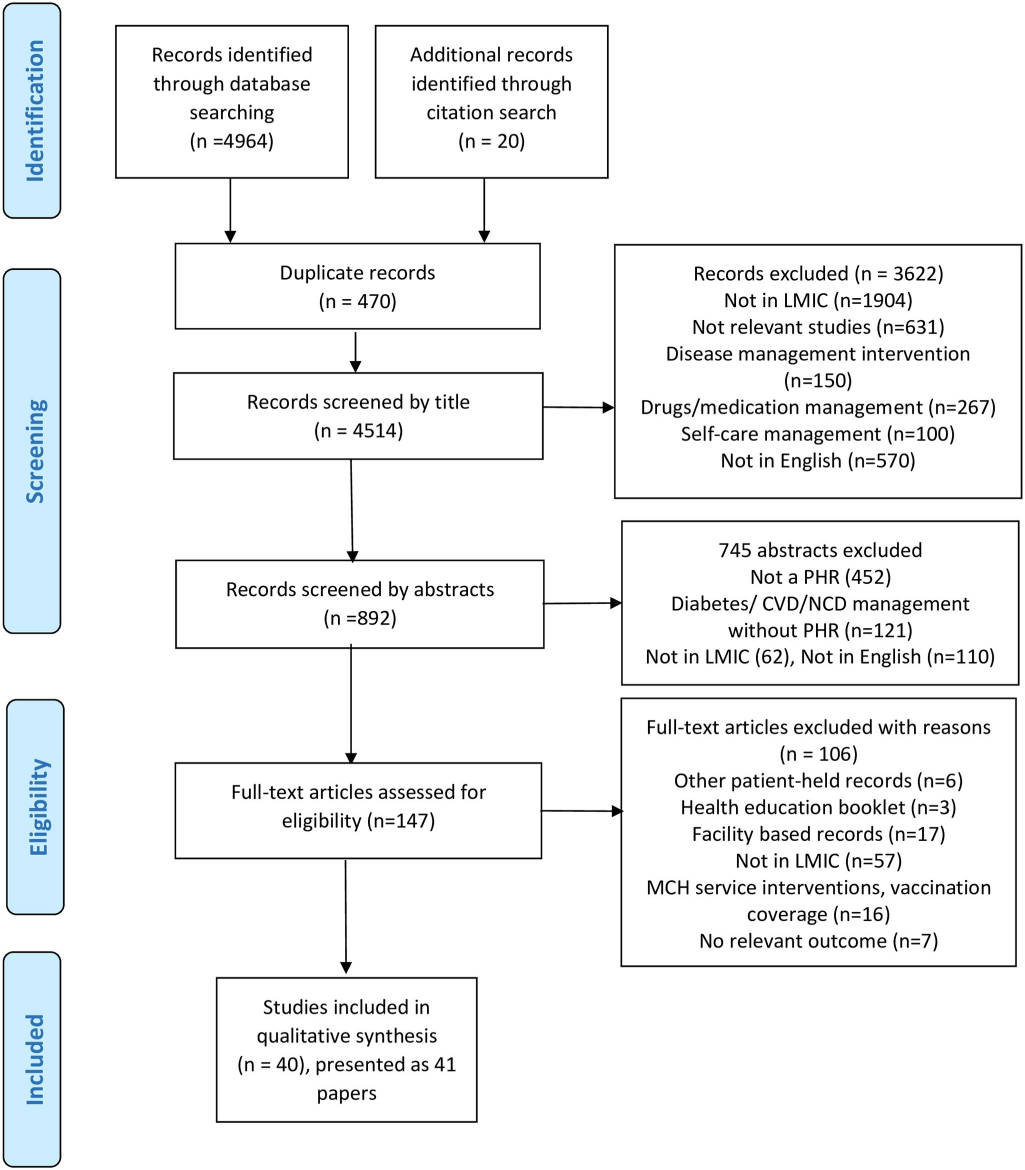
Preferred Reporting Items for Systematic Reviews and Meta-Analyses (PRISMA) flow diagram. LMIC = Low and middle-income countries, CVD = Cardiovascular disease, PHR = Patient-held health record, NCD = Non-communicable disease, MCH = maternal, and child health.

The database searches (MEDLINE, EMBASE, CINAHL, and GIM) found 4964 potential studies of which 470 duplicate studies were removed (Fig 1). Additionally, 20 studies were included from the citation search. In total, 4514 titles were screened for eligible studies. Further, 892 abstracts were assessed for inclusion in the review. Finally, 41 full-text articles (40 studies presented as 41 papers) were included in the review after assessing 147 full papers. We discarded the remaining 106 articles due to the following reasons (a) not being a record for maternal and childcare (n = 9) other MCH interventions such as health education classes, training for birth attendants, etc. (n = 16), (b) not being used in LMICs (n = 57), (c) facility-based health records (n = 17) and (d) no relevant outcome data (n = 7).

### Characteristics of included studies

In total, 407 studies were included and presented as 418 papers. Two were randomised controlled trials [21, 22]; three cluster randomised trials [23–25] and one reporting three year follow-up results of a cluster randomised trial [26]; eight pre-post studies [27–34]; 25 cross sectional studies [35–59]; two qualitative studies [60, 61] and one mixed method study [62]. Sixteen countries; Bangladesh (n = 2) [28, 59]; Brazil (n = 10) [45, 49, 50, 53–57, 60, 61]; Burkina Faso(n = 1) [44]; Burundi (n = 1) [30]; Cambodia (n = 1) [31]; The Gambia (n = 1) [62];Indonesia (n = 7) [23, 25, 34, 36, 46, 47, 52]; Kenya (n = 3) [39, 40, 48]; Lebanon(n = 1) [43]; Malawi(n = 1) [58]; Mongolia(n = 3) [24, 26, 35]; Nigeria (n = 1) [51];Pakistan(n = 2) [21, 22]; Palestine(n = 2) [29, 37]; South Africa (n = 4) [32, 38, 41, 42] and Viet Nam(n = 1) [27] were represented in the included studies. One study reported the use of MCH handbooks in multiple countries (Egypt, India, Pakistan, Philippines, Senegal, Sri Lanka, Yemen and Zambia) [33]. The study characteristics of included studies are provided in Online supplement (S2 Table in S1 File).

### Quality of included studies

Overall, the studies included in the review were moderate to high quality. All randomised controlled trials reported randomisation. However, one cluster-randomised trial did not mention the method of randomisation [25]. The follow-up results of the Mongolian trial [26] reported baseline imbalances between intervention and control groups and differences in follow-up numbers. (Online supplement S3 Table in S1 File). None of the trials (n = 5) [21–25] described outcome assessor blinding. Most pre-post studies (n = 8) described the measurements and adjusted for confounders (n = 4) [29–31, 34]. However, the participants’ representativeness of the target population could not be ascertained in five studies [28–30, 32, 33] due to inadequate descriptions of inclusion and exclusion criteria. Overall, twenty cross-sectional descriptive studies described the sampling strategy adequately [35–37, 39, 42–45, 48–59] and 13 studies described the measuring instruments [35–39, 41–48]. However, the instrument validity and data relevant to assessing non-response bias were not well described in any included studies. Qualitative studies [60, 61] were of medium to high quality. The mixed methods [62] study did not describe the integration of qualitative and quantitative data.

### Summary of results from included studies

The major functions of HBRs, which were evaluated in the included studies, pertained to the recording of health care received by women/children by HCPs and the communication between HCPs and women/family during the health facility visit. In contrast, there were very few studies evaluating referral or information transfer across healthcare facilities/providers. Studies suggested that HBRs could be used to improve handover communication between healthcare visits for HCPs and from HCPs to women/families.

### Functions of HBRs

Of the 41 studies, 30 evaluated the HBR as a tool for recording health services given by HCPs (Table 2). Only six studies evaluated the HBRs as a tool for a referral or for information transfer across healthcare facilities and eight reported on HBRs as a tool for communicating between HCPs and women/families.

**Table 2:** Functions of HBRs evaluated in the included studies.

Function of the HBR	Outcomes pertaining to the function	Studies assessing the function
**Handover communication and information for HCPs at visits**	Availability of HBRs at clinic visits	Tarwa et al. 2007, Brown et al., 2018, Vierira et al. 2009, Palombo et al., 2014
**As a tool for recording healthcare data for HCP**		
	Quality of information recorded (HCP capacity)	Harrison et al., 1998, Vierira et al., 2009, Brown et al. 2018, Ramraj et al. 2018, Naidoo et al. 2018, Kabore et al. 2020, Camargos et al. 2021, Gustaffasson et al. 2020, Wallace et al. 2019, Abud and Gaiva 2015, Adedire et al. 2016, Araujo et al. 2017, Amorim et al. 2018, CoeIho et al. 2021
**Monitoring by HCPs/tracking vaccination status for immunisation programmes**	Healthcare service utilisation and follow-up	Usman et al. 2009, Usman et al. 2011, Mori et al. 2015, Osaki et al. 2019, Wallace et al. 2019, Kaneko et al. 2017, Shah et al. 1993, Aiga et al. 2016, Hayford et al. 2013, Jahn et al. 2008, Yanagisawa et al. 2015, Bhuiyan et al. 2006, Mudany et al. 2015, Osaki et al. 2013, Kitabayashi et al. 2017, Kusumayati and Nakamura 2007, Adedire et al. 2016
**As a tool for referral and information transfer to other HCPs/healthcare facilities**	Availability of HBRs at hospital visits as referral documents.	Gustaffasson et al. 2020, Osaki et al. 2019,
		Bhuiyan et al. 2006, Shah et al. 1993,
		Gonzalez et al. 2019, Camargos et al. 2021,
	Quality of information recorded	Gustaffasson et al. 2020, Gonzalez et al. 2019,
		Camargos et al. 2021
**Monitoring by HCPs**	Health outcomes	Mori et al. 2015, Osaki et al. 2019, Dagvadorj et al. 2017
**Handover communication and information for patients/families**	Knowledge, attitude and practice	Mori et al. 2015, Osaki et al. 2019, Aiga et al. 2016, Yanagisawa et al. 2015, Bhuiyan et al. 2006,
**As a tool for communication from HCPs to patients**		Hagiwara et al. 2013, Kawakatsu et al. 2015, Baequni et al. 2016

### Handover information tool for HCPs

#### Availability or carrying of HBRs to regular health visits, referrals, or consultations

Seven studies measured the availability of HBRs at health care facilities using direct observation (n = 5) [41, 45, 48, 56, 57] and women/caregivers’ self-reported practice (n = 2) [23, 28]. The reported availability of HBRs in health care facilities were 56% (156/300), 76% (516/677), 49.4% (727/1471), 60% (5609/9917), 51% (185/358), 54.6% (100/189) and 83.3% (199/240) respectively.

A Gambian study [62] explored HCPs’ views on the availability and use of documented information in the HBRs and showed that the majority of women (235/250, 94%) brought HBRs in the form of government-issued cards to the hospital at delivery. This Gambian study reported facilitators for using HBRs; these were women acting as agents who transport documented information, clear documentation, and having a designated space on the HBR for referrals. The reported barriers were little or no information being recorded, illegible handwriting, and loss of reports such as lab reports leading to gaps in information in HBRs.

Three studies reported that women/families carried HBRs across healthcare facilities, without measuring the availability at all health care facilities [23, 28, 33]. In a multi-country study [33] that examined documentation in HBRs at referral centres/hospitals, HCPs did not always write the actions they had taken in the HBRs. However, in the Philippines, the staff were oriented about the use of HBR for referrals and 92.4% (n = 66) of HBRs had information documented.

Two studies [41, 48] explored women/caregivers’ reasons for not carrying HBRs to healthcare. For example, (106/300) [41] and (41/129) [48] participants in South Africa and Kenya participants did not know the importance of HBR in health care visits.

### Completeness of relevant fields

Seventeen studies [32, 38, 42, 44, 45, 48–51, 53–59, 62] examined the completeness of fields in the HBRs. Completeness was assessed using different measures (Table 3) and the fields in the HBRs were not uniform across all the reported studies. Of the 17 studies, HBRs were least assessed for completion of maternal parameters (n = 6) and most assessed for child health parameters (n = 15). The reported completeness across different studies and fields in the HBRs varied (Table 4). The most documented information was demographic details and vaccination history [32, 38, 48]. The least documented information was child’s vision problems [48], growth monitoring and vitamin A administration [38, 48], maternal HIV status [38] and doctors’ notes [32] (Table 4).

**Table 3:** Completeness of outcome measures and relevant results in the included studies.

Author, year	Outcome measure defined	Relevant results
Harrison et al., 1998	Percentage of vaccination status for polio, BCG, DTP, Hepatitis-B vaccine, and Measles, charts completed, and notes from doctors, staff, and mothers.	The South African study in 1998 reported 92.5% (419/453) recording of polio vaccination (most) and the least recorded the form of doctor’s notes 11.9% (54/453).
Jahn et al., 2008	BCG Vaccination data	The Malawian study found that vaccination HBRs were available for 63% (3440/5418) children. In the case of BCG data, of the 3487 children under five, 143 documents had no record of BCG (976 children did not have a vaccination card and 2368 cards had documentation of BCG in them).
Vieira et al., 2017	Height and weight of the child	The Brazilian study found that health professionals recorded at least two weight (68.9%) and height (47.3%) measures.
Hayford et al., 2013	Vaccination data from HBR, facility-based records, and maternal recall.	913 children had facility-based vaccination data available; of which 800 children had vaccination HBRs. The measles vaccination coverage based on the mother’s recall was 93.4% (853/913) while HBR data showed 87% (790/913).
Palombo et al., 2014	Demographic, anthropometric measurements, vaccination, growth (height and weight), and development data	The Brazilian study reported that the vaccination schedule was completed in 97% (169/185) of the HBR, but only 9% (14/185) and 8% (13/185) of the HBR, respectively, contained growth charts and properly completed developmental milestones.
Abud and Gaiva, 2015	The development curve was considered complete when 2 or more of the items in the handbook had been filled in (out of 4) and growth data was considered complete when the handbook included at least one weight input every three months, with a minimum of 4 records in the first year of life.	The Brazilian study evaluated the child health handbook for completeness of growth and development data. 95.4% of the 929 handbooks had incomplete missing information related to the development and 79.6% (726/950) had missing or incomplete data in the growth chart
Adedire et al., 2016	Vaccination data in HBRs	The Nigerian study evaluated HBRs to assess vaccination coverage found that 57.9% (275/475) of the children were fully immunised while 42.1% (200/475) were partially immunised while mothers’ recall data found that 74.4% (558/750) of the children were fully-vaccinated, 20.8% (192/750) were partially-vaccinated indicating a poorer recording of vaccination status in HBRs.
Araujo et al., 2017	A score using completed data in growth charts, records of iron and vitamin A supplementation and notes on immunisation schedules.	The study reported adequate data entry in 42% (110/316) HBRs. The least documented data was for iron supplementation and the body mass index-versus-age chart.
Amorim et al., 2018	Completion of fields in HBRs	The study reported 44.5% of the HBR had ≥ 60% of the items completed. The items that should be recorded in maternity wards, birth weight showed the highest proportion of completeness (64.5%); for those that should be filled in PHC/other services, records of vaccines (94.0%) presented the highest completeness in the HBRs.
Brown et al., 2018	Background demographic information, vaccination history, receipt of vitamin A, growth monitoring, early eye or vision screening, and new-born delivery information.	The Kenyan study found that demographic information and vaccination history were recorded in 80% of handbooks. The least documented information was child’s vision problems, growth monitoring, and vitamin A, with entries logged in these fields for 33%, 88%, and 60% of records.
Ramraj et al., 2018	A composite measure of completeness by using the following; infant birth weight, BCG immunisation, maternal HIV status, and an indication of maternal syphilis testing.	Another South African study comparing two cross-sectional surveys found an increase in recording of four areas (infant birth weight, BCG immunisation, maternal HIV status, and indication of maternal syphilis testing) from 23.1% (95% CI = 22.2–24.0) in 2011–12 to 43.3% (95% CI = 42.3–44.4) in 2012–13.
Naidoo et al., 2018	HIV-related completeness, sociodemographic completeness, and neonatal completeness	The South African study, which compared completeness in the card vs book, reported the most completed areas as demographic information, the weight of the child, immunisation, and Vitamin A supplementation in 80% of HBRs in the form of a book. The least documented area was HIV- related information; 24% of HBRs did not have any record of the mother’s HIV status
Gonzalez et al., 2019	Completion of fields in HBR	The Brazilian study reported a completion pattern in the available HBRs. At least 95% HBRs had the following items completed-date of the last consultation visit, maternal height and blood pressure verification, uterine height, foetal heart rate, and the Rh factor; 85% or more: date of the last menstruation, urine test results, and less than 30%: performance of clinical breast examination and cytopathology of the uterine cervix.
Kabore et al., 2020	17 vaccine doses	The Burkina Faso study compared recording in HBRs vs facility-based records using rotavirus and pneumococcal vaccination and found that 80% (492/615) of HBRs were unrecorded for children who had been vaccinated according to facility-based records.
Camargos et al., 2021	Legibility and completeness of sociodemographic, clinical, obstetric, and laboratory data.	The Brazilian study in 2020 evaluated the completeness of ANC records. Clinical parameters such as gestational age 98.4% (388/394) blood pressure 99.4(392/394), fundal height97.7 (385/394), the weight of the mother 98.4% (388/394), etc. were recorded while the least recorded information was on the presence of oedema 44% (174/394). No data was available for centres where care was sought such as the basic reference unit 88% (349/394) maternity unit 76.9% (303/394), and health centre where ANC care82.4% (325/394) was provided. The least recorded data were lab reports.
Gustaffasson et al., 2020	Based on WHO referral criteria- name, age, address, parity, gestational age, complications in the antenatal period, relevant past obstetric complications, treatments applied so far results of those treatments.	The Gambian study reported that HBRs were incomplete with at least one unfilled category 80.1% (189/236). Only 26.7% (63/236) noted the ‘Estimated Date of Delivery’ in the HBRs. Of the 94.2% (97/103) of high-risk women who brought the HBR to admission for delivery only 29.9% (29/97) had their status recorded as high-risk on their card.
Coelho et al.,2021	Completion of development items in the child health handbook	The most recorded item in the handbook was vaccination data 81% (18/22). BMI (Body Mass Index) was not recorded in 72% (16/22) handbooks.

BCG = Bacillus Calmette–Guérin vaccine, DTP = Diphtheria, tetanus and pertussis vaccine, HBR = home-based records.

**Table 4:** Impact of HBR on health service outcomes.

Outcome measured	Author	Relevant results
Health service utilisation	Usman et al., 2009	Trial in Pakistan reported improvement in the 3rd dose of diphtheria-tetanus-pertussis (DTP) vaccination status for children in the intervention arm with HBR and education. There was a 31% (adjusted RR = 1.31, 95% CI 1.18–1.46) increase in uptake of 3rd dose DTP in 2009.
	Usman et al., 2011	Trial in Pakistan with HBR for vaccination. There was a 67% (RR = 1.7; 95% CI = 1.4, 2.0) increase in uptake of 3rd dose DTP in 2011.
	Wallace et al., 2019	Another trial in Indonesia with HBR and a reminder sticker for parents demonstrated that children in the HBR and sticker group were 50% more likely to receive a third dose of a vaccine containing diphtheria, tetanus, pertussis, hepatitis B, and *Haemophilus influenzae* type b antigens (DTPcv3) within 60 days of DTP1 (RR = 1.46, 95% CI 1.02–2.09).
	Mori et al., 2015	The trial in Mongolia reported that women in the intervention group attended antenatal clinics more than the control group (RR = 1.158, 95% CI 0.876–1.532) p-value = 0.30*.
	Osaki et al., 2019	While the trial in Indonesia found that after using HBR, women were more likely to receive two doses of tetanus immunisation, visit ante-natal clinic four times, professional assistance during child delivery and ensure that their children took vitamin A supplements (OR = 2.03, 95% CI: 1.19–3.47).
	Aiga et al., 2016	Demonstrated a significant increase in the proportion of pregnant women who received at least three antenatal visits to the clinic from 67.5% (540/800) in pre-intervention to 92.2% (747/810) in post-intervention (P < 0.001).
	Yanagisawa et al., 2015	The intervention increased ANC attendance(4 times increase), delivery with SBAs (Skilled birth attendants), DID (difference-in-differences) = 12.2 (OR = 2.613, p<0.01, AOR = 1.092) and delivery at a health facility DID = (OR: 2.499, p<0.01, AOR = 1.866) even after adjusting for maternal age, education and economic conditions.
	Bhuiyan et al., 2006	Improved antenatal clinic use in the post-HBR group (55.9% vs 35.5%, p<0.05)
	Kaneko et al., 2017	Found that after the introduction of an MCH (maternal and child health) handbook post-natal coverage improved from 43.9% in 2013 to 54.2% (p < 0.05) in 2014.
	Shah et al., 1993	Use of HBR improved ante-natal and post-natal clinic visits in the Philippines and Zambia, with mothers explaining in focus groups that they felt that their clinic attendance had improved and that they perceived themselves to receive better care.
	Mudany et al., 2015	In Kenya HIV DNA testing in infants rose from 27 000 in 2007 to 55 000 in 2010 to 60 000 in 2012, which represents approximately 60% coverage of estimated HIV-exposed infants.
	Kitabayashi et al., 2017	The Palestinian survey found that mothers with HBR had significantly higher odds of receiving all three medical tests (aOR 1.58; 95% CI 1.287–1.932) and of having been informed about five or more health education topics (aOR 2.10; 95% CI 1.746–2.534) as part of antenatal care, (adjusted for age).
	Osaki et al., 2013	A repeated cross-sectional study reported that using an HBR was associated with a 3 times higher probability that the mother would use a skilled birth attendant (95% CI 1.031–9.477). Mothers reading most or all of the HBR was found to be associated with mothers receiving ANC at least 4 times (OR = 1.736; 95% CI 1.194–2.522) and with their receiving at least two TT (tetanus toxoid) immunisations (OR = 1.576; 95% CI 1.146–2.166).
	Kusumayati and Nakamura, 2007	Having HBR was associated with having a delivery assisted by trained personnel [adjusted odds ratio (aOR): 2.12, 95% confidence interval (CI): 1.05 4.25], receiving maternal care (aOR: 3.92, 95% CI: 2.35 6.52), completing 12 doses of child immunisation for seven diseases (aOR: 4.86, 95% CI: 2.37 9.95), and having immunisation before and after childbirth (aOR: 5.40, 95% CI: 2.28 12.76).

HBR = home-based records, AOR = adjusted odds ratio, OR = odds ratio, HIV = Human Immunodeficiency virus, DNA = deoxyribonucleic acid,

*not statistically significant.

In a study conducted in Burkina Faso [44], comparing recording in HBRs versus facility-based records, 80% (492/615) of HBRs were unrecorded for children who had been vaccinated according to facility-based records. A Gambian study [62] reported that HBRs were incomplete with at least one undocumented category for 80.1% (189/236) of mothers. Further, 26.7% (63/236) noted the ‘Estimated Date of Delivery in the HBRs. Of the 94.2% (97/103) of women who brought the HBR to admission for delivery, only 29.9% (29/97) had their status recorded as high-risk on their HBR. A study in Lebanon [43] assessing the legibility of handwriting in HBRs found that one in five cards (90/460) was scored as ‘poor’.

### HCPs’ perceptions on the usefulness of HBRs

Five studies [25, 29, 31–33] explored the usefulness of HBRs in providing health education to women/parents by providers. Two qualitative studies found that HCPs perceived families did not use HBRs for care optimally [60, 61]. HCPs reported HBRs as a useful tool for giving them confidence in providing health education [29, 31, 33] helping them to identify risks [33], and reminding the women/families regarding vaccination [25]. In a South African cross-sectional study [32] assessing the acceptability to nurses of a maternal and child handbook over a card, nurses were satisfied with the health information (95.6%), immunisation information (94.7%), weight charts (89.5%), and notes for both staff (91.3%) and mothers (93.9%) in the handbook.

#### Health service utilization/uptake of services

The majority of included studies (n = 14) evaluated the effects of HBR on healthcare service utilization. The three key areas of measurement of services were vaccinations, antenatal clinic visits, and a skilled birth attendant for delivery. Two trials in Pakistan [20, 21] reported a 31% (adjusted RR = 1.31, 95% CI 1.18–1.46) and 67% (RR = 1.7; 95% CI = 1.4, 2.0) improvement in the 3^rd^ dose of diphtheria-pertussis-tetanus (DTP) vaccination status for children in the intervention arm with HBR and education in 2009 and 2011 respectively. Another trial in Indonesia [25] also reported a 50% improvement in timely vaccination of 3^rd^ dose of DTP among those with HBR and a reminder sticker for parents.

Five studies demonstrated an improvement in antenatal clinic visits [23, 27, 33, 47]. Detailed outcomes are summarised in Table 4. A trial in Mongolia [24] showed that women in the intervention group attended antenatal clinics more than the control group (RR = 1.158, 95% CI 0.876–1.532) p = 0.30. Another trial in Indonesia [23] found that after using HBR, women were more likely to receive two doses of tetanus immunisation, visit the ante-natal clinic four times, have professional assistance during child delivery and ensure that their children took vitamin A supplements (OR = 2.03, 95% CI: 1.19–3.47). In a repeated cross sectional study in Indonesia the use of HBR was associated with a 3 times higher probability of using a skilled birth attendant by mother (95% CI 1.031–9.477) [36]. Further, mothers with HBR were likely to receive more medical tests [37, 41], post-natal care [31, 34] and health information from HCPs.

#### Maternal, neo-natal, and child health outcomes

In a Mongolian trial [24], complications in maternal health were more likely to be identified, with maternal morbidity during pregnancy at 12.3% in the intervention group compared with 5.7% in the control group (p-value = 0. 01). However, HBRs had no effects compared to the unspecified pre-existing system in the control group on neonatal death or stillbirths (RR 1·0 95% CI: 0·99–1.01, p = 0.512). In the three-year follow-up results in the Mongolian trial [26], small but significant reduced risk of cognitive development delay in children (OR 0·32, 95% CI 0·14–0.73, p-value = 0.007) was reported. A trial in Indonesia [23] reported lesser risk of underweight children (OR = 0.33, 95% CI: 0.12–0.94; p<0.05) or lesser risk of children with stunted growth (OR = 0.53, 95% CI: 0.30–0.92; p<0.05) for caregivers using HBR.

### Handover communication and information tool for women/caregivers

#### Knowledge, attitude, and practice

Ten studies [23, 24, 27–29, 34, 46] evaluated the use of HBR and its effects on knowledge, attitude, and practice outcomes. One study did not report any improvement in knowledge after the use of HBR [46]. Individual study results can be found in Table 5. In general, trials (n = 2) found an improvement in complementary feeding [23] and the adoption of healthy behaviours by family members of pregnant women [24] after using HBR. Five pre-post studies [27–29, 31, 34] assessed the knowledge, attitudes, and practices of mothers/caregivers after the introduction of HBR. They showed an improvement in knowledge and practice of breast feeding [27–29, 31, 34], awareness of danger signs in pregnancy [28, 29], and awareness of childhood illness [31].

**Table 5:** Handover communication tool from HCPs to women/families.

Outcomes measured	Studies which measure the outcome	Relevant results
Knowledge, attitude, and practice	Mori et al., 2015	The majority of women did not drink alcohol (7.9% in the intervention group compared with 14.1% in the control group, p = 0.161), and approximately half of family members stopped smoking at home (51% in the intervention group compared with 60% in the control group, p = 0.048).
	Osaki et al., 2019	Reported an improvement in the initiation of complementary feeding at 6–9 months OR 4.35 (2.85–6.65) p = 0.001.
	Aiga et al., 2016	The knowledge and practice of exclusive breastfeeding improved from 66.1% in pre-intervention to 86.7% in post-intervention (P < 0.001) and from 18.3% in pre-intervention to 74.9% in post-intervention (P < 0.001) respectively.
	Bhuiyan et al., 2006	Reported increased awareness of breastfeeding in the intervention group (28.7% of cases and 4.6% of controls (no p-value)), improved awareness of danger signs of pregnancy (46.9% case and 5% control groups (no p-value)), and knowledge of recommended ante-natal care (78% case and 8.3% control groups, p < 0.05).
	Hagiwara et al., 2013	Reported statistically significant improvement in awareness of breastfeeding for literate women (t-test = 1.85, p ≤ 0.1), awareness of rupture of membranes (t-test = 2.04, p ≤ 0.05) and knowledge of family planning among literate women (t-test = 3.16, p = 0.01).
	Yanagisawa et al., 2015	Evaluated the impact of the handbook by using difference-in-differences (DID) analysis and found that the intervention group had improved awareness of childhood illness (R = 6.2 points for anaemia, 9.9 for parasites, 7.5 for HIV transmission), knowledge of breastfeeding R = 6.2 for early breastfeeding (no p-value) and improved awareness of danger signs of pregnancy.
	Baequni et al., 2016	Overall, compared with the control group, the home-based records group had more knowledge and better practices during pregnancy, delivery, and child health care (e.g., immunisation).
	Kawakatsu et al., 2015	Reported that possession of an HBR was associated with higher health awareness (AOR: 1.41; 95% CI 1.138–1.724; P = 0.002).
	Nasir et al., 2017	Reported that attending mother class using HBR (intervention) significantly increased knowledge of breastfeeding initiation and hepatitis B immunisation (p<0.05). Mothers in the intervention group had the likelihood of practicing good new-born care compared with the control group (odds ratio: 1.812; 95% confidence interval: 1.235–2.660).
	Tjandraprawira et al., 2019	Reported no improvement in knowledge scores for women.

HBR = home-based records, AOR = adjusted odds ratio, OR = odds ratio.

#### Usefulness of HBRs to mothers/caregivers

Fives studies [27–29, 31, 35] assessed the utility of HBRs to mothers/caregivers. Mothers could read the contents of HBR [28, 35], discuss with husbands/partners [29, 31], and record breastfeeding practices in the HBR [27, 35]. In Viet Nam [27], it was found that 68.1% of HBRs (MCH Handbooks) had check-boxes on exclusive breastfeeding ticked by mothers. Focus group discussions with mothers clarified that they carried the records to all healthcare visits but often forgot to record breastfeeding practices in the HBR. In a multi-country study [33], based on focus group discussions, many mothers said that the HBR was a useful "passport" in the referral system because it led to contact with "someone who knows our problem and takes better care of us at the centre".

## Discussion

### Summary of findings

We have summarized the available evidence on HBRs from low and middle-income countries for women/families in this systematic review; specifically to improve the informational continuity for providers and for communication between HCPs and between HCPs and women/families and outcomes of using HBRs. In general, findings suggest HBRs are under-utilized as tools for improving information availability for HCPs across health care facilities in LMICs. Overall, there is sub-optimal recording in HBRs with exception of vaccination data. However, we have shown that HBRs may facilitate improving healthcare services uptakes such as improved antenatal clinic visits, immunisation rates, and a delivery attended by skilled healthcare personnel in LMICs. Further, there was a modest impact on maternal and child health outcomes due to the use of HBRs. The review suggested that HBRs might facilitate the detection of the risk of pregnancy complications by HCPs and have a protective effect on the cognitive development of children, possibly by early detection of developmental delays. Improved awareness of breastfeeding practices and danger signs in pregnancy amongst mothers were reported after using an HBR.

This review found the frequency with which LMIC women/caregivers carry records to healthcare visits varies. However, one of the recent studies [62] reported almost 94% of women had documentation with them at the time of delivery. This finding is consistent with reviews done in high-income countries with most women/caregivers bringing their notes to clinic check-ups [12, 14, 63]. The retrieval of information from poorly maintained facility-based registers in many LMICs presents an opportunity to use HBRs as a surrogate tool for documented handover information [64]. Women/caregivers who did not carry records to visits were found to be unaware of the importance of having recorded at each visit [48]. However, when HCPs insisted on having the HBR, women/caregivers brought them to visits [25]. Thus, HCPs reminder creates more awareness among women/caregivers of carrying records and thus improve the availability of records at health care visits. Additionally, women carrying HBRs to all visits, irrespective of the reason for the visit, can enable better informational continuity for HCPs, subject to all HCPs being trained to enter minimal data in them.

Although HBRs are intended to be used as complementary documents to facility-based records, the availability of HBRs at healthcare visits enables better clinical decision-making [65]. However, from this review, the overall documentation in the HBRs ranged from 11% to 92%. The least utilized fields were doctors’ notes, child growth, and development data, maternal HIV status, and high-risk pregnancy status. However, there are not enough data from these papers to conclude reasons for poor data entry by HCPs. To be useful for all HCPs, HBRs must be completed accurately and capture the necessary clinical information in a standardized manner. The Mongolian trial showed evidence of HBRs facilitating detection of pregnancy complications [24]. However, the limited evidence on medical information availability for HCPs in hospitals or secondary care shows that for HBRs to be useful for handover, referral using HBRs must be an established practice [20]. These results indicate the need for better training for HCPs to ensure better utilization of these records. This could be most useful in pluralistic health systems, where referral pathways are not implemented or adhered to and when patients/families self-refer themselves to different HCPs. Additionally, HBR stock-outs [66] and fragmentation of information due to multiple HBRs per patient [67] act as barriers for recording and maintaining HBRs as an established practice. Therefore, it is necessary to consider the best way to implement such a programme and if there can be a secure system for keeping it going in a way that allows for all to have one HBR and for every HBR to have the full and latest information on it. Otherwise, the cost-effectiveness and benefits of the system are questionable.

The evidence suggests that healthcare utilisation is improved with HBR in LMICs. This result is consistent with previous findings from prior systematic reviews. Magwood et al. found that HBRs had statistically significant effects on improving antenatal care attendance; however, the evidence base was small and of low to very low certainty [14]. The mechanism of improvement of healthcare utilization is unclear. This is because the RCTs [21, 22] showing improvements in vaccination uptake demonstrated that this was due to HBR intervention and verbal reminders from HCPs. Therefore, whether having the information in the HBR is functioning as a trigger for HCPs to provide verbal reminders and health information, or whether patients’ engagement with the records is acting as a reminder, is not well established. This is an important research gap; addressing it will enable alignment of the content of the HBRs to the literacy levels of parents or HCPs and with the function, it serves each user group. Overall, the results obtained (such as improvements in vaccination uptake) from relatively minor studies from specific geographical settings make it difficult to generalize the findings to all regions in LMIC and suggests lower certainty of the outcome.

From the review, HCPs find HBRs useful for health promotion and healthcare communication with women/families. However, limited studies [29, 33] reports on HBRs improving the communication of women/caregivers with HCPs. This finding contrasts to studies in HICs, which suggest that HBRs enhance women’s feeling of empowerment and thereby enable better communication with HCPs [5, 12]. Additionally, there is limited evidence for pregnant women reading and recording data in HBRs from the review. Therefore, further studies, which explore the mechanisms by which behaviour change occurs with HBRs, are necessary to determine the type of health education material in them [16]. However, there is modest evidence of HBRs improving communication with husbands/partners/families. This finding is similar to a qualitative review by Magwood et al., which suggests HBRs improved engagement with care for husbands and families [15]. Therefore end users’ involvement in re-designing HBRs may enable better engagement from women/families (particularly with lower literacy) and providers [68]. A user-centred design could overcome the needs and facilitate a better understanding of the users [16].

#### Implications for research and practice

Our findings highlight the need for engagement with end users such as frontline workers, health care providers, patients and families while designing an HBR. Additionally in those LMICs with existing HBRs, implementation evaluations of HBR use and re-formatting or re-designing the content of HBR to meet the needs of end-users will enable better use of HBRs in routine practice. Further research is needed to understand the unused content in HBRs.

#### Strengths and limitation

The comprehensive search strategy for, and focused review of, studies in LMICs has helped to understand the usefulness of HBRs in that setting. However, the inclusion of studies in the English language alone may have excluded studies in regional languages. A possible limitation of this review is in the categorisation of the included studies as handover communication for HCPs and patients/families. This is because most studies contain results, which can fit into both categories. For example, the detection of cognitive delay may be due to HCPs recording and monitoring growth and parents being aware of the milestones and communicating this during healthcare visits to HCPs. Therefore, a clear distinction of the function of HBRs for HCPs alone, particularly in the absence of trials focused on the improvement of information available for providers, could not be made, calling for more research to enable an improvement in continuity of information and maternity care. This review has not evaluated the content and design quality of HBRs that may hinder the utility of HBRs for providers. However, in assessing the completion of records, it was found that the fields were different for HBRs from different countries. Therefore, having a minimum criterion, which is necessary for handover across HCPs will enable future studies to assess the quality of documented information. Additionally, this review did not capture the different health education/promotion messages included in each of HBR and its appropriateness, which can affect the MCH outcomes measured.

## Conclusion

Overall, there is a lack of studies from LMICs examining the use of HBRs in improving health outcomes, which is consistent with previous systematic reviews. The findings from the review demonstrate a lack of literature on the availability of HBRs for secondary encounters such as referrals. Our study extend the evidence on current use of HBRs in LMICs for improving informational continuity for HCPs. Although there is a great potential for HBRs to enable information transfer for safe continuity of maternal and child care, evidence suggests that data completeness in the HBRs is currently suboptimal (except vaccination data) leading to decreased informational continuity for HCPs in LMICs. The review supports the HBR [potentially] being an effective tool for handover communication from HCPs to women/caregivers, in improving utilisation of antenatal visits, immunisations, and skilled birth delivery in LMICs similar to the findings from previous reviews. There is some evidence on improving mothers’ knowledge of breastfeeding and identification of danger signs in pregnancy.

## Supporting information

S1 File S1 Table: Functions of home-based records (HBR), S1 Box: Different types of HBRs included in the review, S2 Table: Characteristics of included studies, S3 Table: Results of mixed methods appraisal tool, S4: Systematic review protocol registered in PROSPERO (CRD42019139365), S5: Example search strategy in EMBASE, S6: PRISMA checklist, S2 Box: Search strategy in Ovid MEDLINE. (DOCX)

S1 Checklist (DOCX)

## Abbreviations

HBR: Home-based records
HCP: Health care providers
LMIC: Low and middle income countries
MCH: maternal and child health
