# Supplementary material for: A systematic review of home-based records in maternal and child health for improving informational continuity, health outcomes, and perceived usefulness in low and middle-income countries
Source: PLoS One. 2022 Aug 4;17(8):e0267192. doi: 10.1371/journal.pone.0267192 (PMC9352021; doi:10.1371/journal.pone.0267192)
Supplement: S1 File — (DOCX) [file pone.0267192.s001.docx]

Online supplemental

Table S1: Functions of home-based records (HBR) as described by Osaki et al and Brown et al

| Osaki et al ^[[1]](#footnote-1)^ | Brown et al ^[[2]](#footnote-2)^ | Handover communication function for the review |
| --- | --- | --- |
| Data recording and storage (serve as the reliable documented source of individuals’ health data) | Tool for documenting vaccinations and other primary care services, particularly during childhood but increasingly across the life course, in a standardized manner. | Handover communication across healthcare visits |
| Monitoring and referral in HBRs enable healthcare workers, correctly and efficiently track the personal health data and treatment histories of clients. | When appropriately completed and referenced, HBRs provide necessary information for frontline clinical decision-making that may ultimately improve continuity of care. | Handover communication across health care providers (HCP). |
|  | HBRs complement facility-based record systems and serve as a verified surrogate in the absence of functioning facility-based record systems. | Handover communication across healthcare visits and HCPs. |
| Behaviour change communication (particularly for integrated handbooks) | HBRs help stimulates demand for vaccination services by raising caregivers' awareness of the benefits of vaccines, the recommended vaccination schedule, and the date of the child's next vaccination visit. | Handover communication from HCPs to women/families. |
|  | HBRs serve as a prompt to initiate a discussion between health care workers and caregivers about the importance of immunisation during a health encounter at a facility or an outreach session. | Handover communication from HCPs to women/families. |
| Continuous self-monitoring by mothers and caregivers to recognize and address health risks via self-care or self-referral to a higher or lower level of health facility |  | Handover communication from women/families to HCPs. |
| Table S1 legends; HBR=home based records, HCP=healthcare providers | | |

Box S1 Different types of HBRs included in the review

1. Antenatal records or women-held records- women are given their own case notes to carry during pregnancy.
2. Vaccination cards- Parent or caregiver held vaccination cards for children.
3. Child Health Records- Parent or caregiver held records including growth and development information and vaccination information
4. Maternal and child health handbook- Parent or caregiver held handbooks with both maternal and child health information

Table S2: Characteristics of included studies

| Author, published year | Country | Study type | Objective of study | Type of HBR | Population | Outcomes measured | Relevant Results |
| --- | --- | --- | --- | --- | --- | --- | --- |
| Aiga et al, 2016 | Viet Nam | Pre-post survey and qualitative focus group discussions | To determine pre-post intervention of HBR, levels of pregnant women’s knowledge, attitude, and practices (KAP) towards their antenatal care service utilisation and exclusive breastfeeding practices. | The standardised MCH Handbook was composed of a recording section and guidance section for respective maternal and child health stages, i.e. pregnancy, delivery, postnatal, new-born, and childhood. | Mothers who received the MCH handbook within the first implementation phase. | The knowledge, attitudes, and practice of mothers about antenatal care, breastfeeding practices, and use of the MCH handbook. | The proportion of pregnant women who made three or more antenatal care visits significantly increased from 67.5 % (pre-intervention) to 92.2 % (post-intervention) (P < 0.001). |
| Abud and Gaíva, 2015 | Brazil | Cross-sectional | To analyse the input of growth and development data in the Child Health Handbook. | Child Health Handbook | Mothers or guardians of the children | Completeness of growth and development data in child health handbook | Of the analysed handbooks, 95.4% of the development data and 79.6% of the data in the growth charts were incomplete. |
| Amorim et al, 2018 | Brazil | Cross-sectional study | To describe the filling process of the Child Health Record (CHR) in health care services | Child Health Record(CHR) | Children (3-5 years) resident in Belo Horizonte who carried the 6th Edition of the CHR (2009) | Frequency of filling of each CHR field and the CHR frequency with filling percentage of < 60%, ≥ 60%, ≥ 70%, 80%, and ≥ 90%, an association between the percentage of fields’ filling to be registered at birth or in the PHC/other services, comparison of the number of fields in the CHR completed | A total of 44.5% of the CHR had ≥ 60% of the items completed. The CHR with ≥ 60% and ≥ 70% filling in the registration fields in the PHC/other services were significantly higher when the CHR also showed a higher percentage of filling (≥ 60%) of the registering fields in the maternity ward. This difference was not observed for the largest percentage of fields in the PHC/other services (80 and 90%), by the small number of CHR. |
| Andrade et al, 2014 | Brazil | A qualitative study with a phenomenological approach | To understand the experiences of health professionals in primary care with the Child Health Booklet in child health care. | Child Health Booklet | Health professionals (doctors and nurses) | Experience with the booklet in child health care and filling out of the booklet | The understanding of the booklet of child health as a means of monitoring the growth of children and their vaccination status was the only conception present in the speech of all professionals and even for some, this understanding turned out to be the only value of the booklet in their health practices with the child. |
| Araujo et al, 2017 | Brazil | Cross-sectional | To evaluate the use of child health surveillance tools (by health professionals) especially focusing on growth. | Child Health Handbook | Mothers or guardians of the children | Growth charts, records of iron and vitamin A supplementation, and notes on immunisation schedules registered in the instrument were analysed. | All the factors studied showed high frequencies of limited data entry, ranging from 41.1% for the weight-versus- age chart to 95.3% for the body mass index-versus-age chart. Higher frequency of inadequate data entry was found among children aged 25 months and over and among those living in areas of these municipalities with minimal numbers of professionals in the healthcare teams. |
| Baequni et al, 2016 | Indonesia | Cross-sectional (Secondary data analysis) Data included Indonesia Demographic and Health Surveys (IDHS) | To analyse the effects of home-based records on pregnancy, delivery, and child health care in Indonesia | Maternal and Child Health Handbook (MCHHB) | Women who had children under 5 years old | The effects of home-based records on pregnancy, delivery, and child health care | The home-based records group had more knowledge and better practices during pregnancy, delivery, and child health care (e.g., immunisation). The home-based records group knew how to solve the problems of complications during pregnancy and used skilled birth attendants for delivery. This study also found that husbands in the home-based records group were involved in discussing the delivery location, finding transportation, and identifying a blood donor. |
| Bhuiyan et al, 2006 | Bangladesh | Pre-post study | To develop MCH handbook and to assess its effect on mother’s knowledge, practice and utilization of MCH services. | MCH handbook | Pregnant mothers  Health care providers | Data to develop the content of the handbook was gathered through focus group discussion with mothers and health care providers. Mothers’ knowledge, attitude and practice regarding MCH services were collected through pre-post surveys. | There has been satisfactory improvement of knowledge regarding antenatal care, danger signs, breastfeeding and vaccination among case group after intervention of MCH handbook. |
| Brown D W et al, 2018 | Kenya | Mixed methods-Cross-sectional survey with open-ended questions | To describe HBR ownership and report on the utilization of selected recording areas in HBRs Kenya. | Child HBR/integrated MCH book | The caregivers of children aged 0±23 months exiting selected health facilities and healthcare workers. | The caregivers were asked several questions related to how they obtained and used their child's HBR. The HBRs were checked for their completeness. | One-third (n = 41) of those without an HBR in hand at the visit noted that they did not know the importance of bringing the document with them. Roughly two-thirds (n = 443) of caregivers noted they were asked by clinic staff to see the HBR during the clinic visit. Across the 516 reviewed HBRs, recording areas were most commonly identified for the child's demographic information (80% of HBRs) and vaccination history (82%) with the information marked in>90% records. |
| Camargos et al, 2021 | Brazil | Cross-sectional | To evaluate the antenatal care (ANC) home-based records of puerperal women attended in public and private maternity hospitals. | ANC Mother HBRs | Puerperal women. | Records were assessed regarding completeness, legibility, and completeness of sociodemographic, clinical, obstetric, and laboratory data. | 88.5% of the cards had no record of the name of the primary care unit of reference, 76.9% of the maternity of reference, and 82.4% of the ANC institution. Evaluation of oedema, use of ferrous sulphate and folic acid were not registered in 55%, 91.1%, and 92.6% of the cards, respectively. The absence of records in the weight chart was identified in 86.8% of the cards, and in the uterine height chart, 79.7%. |
| Coelho et al, 2021 | Brazil | Cross-sectional | To identify parents’ knowledge about the Child Health Handbook, map the completion of the booklet by professionals, and correlate the completion of the booklet with the guidance of parents by health professionals. | Child Health Handbook | Mothers or guardians of the children | Completeness and parents' knowledge. | The most recorded item in the handbook was vaccination data 81% (18/22). BMI has not been recorded in 72% (16/22) handbooks. |
| Dagvadorj et al, 2017 | Mongolia | Follow up cluster RCT | To assess the effectiveness of the handbooks on child development in Mongolia. | MCH Handbook | Women living in the Bulgan province of Mongolia who gave birth between March and August 2010 participated in the study and the three-year follow-up if they still lived in the area. | The primary study outcome was a risk of developmental delay as assessed by the Mongolian  Rapid Baby Scale. | In Mongolia, a 3-year follow-up showed a reduced risk of cognitive development delay in children (OR 0·32, 95% CI:0·14–0·73, p-value = 0.007) |
| Adedire et al, 2016 | Nigeria | Cross-sectional study | To assess immunisation coverage rates and to identify the factors associated with vaccination status of children 12–23 months in a rural district in south-western Nigeria. | Vaccination cards | Mothers | Vaccination status of the children | Of the 750 children, 475 possessed vaccination cards, indicating a vaccination card retention rate of 63.3 %. Using mothers’ recall, 558 (74.4 %) of the children were fully-vaccinated, 192 (20.8 %) were partially-vaccinated, and 36 (4.8 %) were non-vaccinated. However, based on immunisation cards, 275 (57.9 %) of the children were fully immunised while 200 (42.1 %) were partially immunised. |
| Gustaffsson et al, 2020 | The Gambia | Mixed-methods study | To assess the number, type, content quality, and completeness of women-held documents on admission to maternity units in The Gambia. To explore context-specific barriers and facilitators to effective use of women-held documents in maternity units by health professionals and maternity staff, especially for women admitted with high-risk pregnancies or deliveries. | Maternity cards | In-patient women aged 16 and over three maternity hospital departments in the Greater Banjul. | Characterised the nature and quality of documents; including the number of each type of document, whether individual criteria were met, and how many women carried documents that met the minimum criteria. | Of the women admitted, all but 10/250 (4%) brought either a maternity card or a structured referral sheet. Women were less likely to have documents complete if they were illiterate and had not attended three maternity appointments. During qualitative interviews, three themes were identified: women as agents for transporting information and documents (e.g. remembering to bring maternity cards); the role of individual healthcare professionals’ actions (e.g. legibility of handwriting); system and organisational culture (e.g. standardised referral guidelines). |
| Hagiwara et al, 2013 | Palestine | Pre-post study | To examine the effect of the MCH handbook on women's knowledge and behaviour. | MCH Handbook | Women who used the MCH centres and healthcare workers of those centres | Knowledge, attitude, and practice related to MCH. User satisfaction and barriers to use of MCH handbooks. | Knowledge related to MCH such as the importance of exclusive breastfeeding and how to cope with the risks of rupture of membranes during pregnancy increased among MCH handbook users, especially among less-educated women. The MCH handbook may be an effective tool for communication with health providers and husbands, for both highly educated and less-educated women during their first pregnancy. |
| Harrison et al, 1998 | South Africa | Cross-sectional | To determine the relevance of the road to health book along with the road to health card. | Road to health book | Nurses and mothers who used the RTH books. | The use of and comments on the RTH book by nurses and mothers were based on analysis of the questionnaires. | Most (81.6%) preferred the RTH book to the Road-to-Health card. It was sturdy (95.6%), had a satisfactory layout (87.5%), and provided useful information for health personnel (71.9%) |
| Hayford et al, 2013 | Bangladesh | Cross-sectional | To compare measles vaccination coverage estimates from surveys (vaccination card/card plus history), clinic records, and immune markers in oral fluid and blood. | Vaccination card | Mothers | Six indicators of measles vaccination history were ascertained for each child:  1) maternal report 2) card record of vaccination 3) ‘card + history’; 4) EPI record of vaccination in clinic books; 5) protective levels of measles IgG antibodies in oral fluid and 6) protective levels of measles IgG antibodies in the blood. | 913 children had facility or clinic-based vaccination data available; of which 800 children had vaccination HBRs. The measles vaccination coverage based on the mother’s recall was 93.4% (853/913) while HBR data showed 87% (790/913). |
| Hikita et al, 2018 | Mongolia | Cross-sectional | To investigate the use of an MCH handbook, and related factors, in Mongolia. | MCH Handbook | Women living in Bulgan Province, Mongolia, with children born between January and December 2010 (and who thus would be 3 years old in 2013) were selected as participants for the study. | Mothers’ utilisation of the MCH handbook was defined based on questions for reading and recording of details in the handbook. | 88.1% of users reported having read the handbook. In this study, two-thirds of participants  who had received an explanation on how to use the MCH handbook reported having made a record in it, whereas only one-third of those who had not been taught how to use it reported having made a record in it. This suggests that receiving an explanation from medical personnel is important  for women to use the handbook. |
| Jahn et al, 2008 | Malawi | Cross-sectional | To assess factors related to recorded vaccine uptake, which may confound the evaluation of vaccine impact. | Parent held health documents | Mother | Vaccination coverage by age | Of 5418 children, vaccination documents were available for review for 3440 (63%). Documents were missing for 37% and exact birth dates for 29% of the 5418 under 5-year-olds in this population. The BCG vaccination data, of the 3487 children under five, 2368 cards had documentation of BCG in them. |
| Kaneko et al, 2017 | Burundi | Pre-post study | To assess the effectiveness of the MCH handbook for increasing notification of birth at health facilities and post-natal care (PNC) uptake. | MCH Handbook | Mothers having infants aged less than six weeks | Socio-demographic status, delivery place by a type of health facility, the proportion of mothers having the MCH handbook, having received notification of birth at a health facility, having delivery mode records, having accurate birth weight data by recall or records, and receiving guidance on PNC. | 95.1% of mothers had an MCH handbook post-study. The observed in the proportion of mothers receiving notification of birth at health facilities, from  4.6% to 61.0% (95% confidence interval [CI]: 55.9%–66.2%), and the proportion of mothers receiving guidance on PNC, from 35.9% to 64.2% (95% CI: 59.2%–69.3%). |
| Kabore et al, 2020 | Burkina Faso | Cross-sectional | To assess the characteristics of HBRs and FBRs, their completion by vaccination providers, and their usefulness in estimating vaccine coverage (VC) | Vaccination HBR | Caregivers of children aged 0 to 23 months | Characteristics and completion patterns of HBR, concordance of HBRs and FBRs to determine their reliability as data sources in estimating vaccine coverage | Half (50.6%) of HBRs were non-standard. About two-thirds (64.6%) of caregivers were concerned with discordant information. Multivariate logistic regression model showed that standard HBR was protectively associated with discordant information (OR = 0.46, 95% CI: 0.26–0.81, p = 0.010). |
| Kawakatsu et al, 2015 | Kenya | Cross-sectional | To clarify the effectiveness of and identify the factors related to possession of an MCH handbook among parents in rural Western Kenya using propensity score matching (PSM)." | MCH Handbook | Mothers with children aged 12-24 months. | Vaccination status, health-seeking behaviour for fever, diarrhoea, maternal health knowledge. | MCH handbook was an effective tool for improving both health knowledge and health-seeking behaviour in Kenya. |
| Kitabayashi et al, 2017 | Palestine | Cross-sectional (secondary data) | To assess associations between MCH  handbook ownership and receipt of selected content of antenatal care services in Palestine | MCH handbook in Palestine was a 60-page booklet written in Arabic, which consisted of a medical record section and a health education section. | Anonymous data set of the Palestinian Family Survey 2010 -data of 2026 women who had live births within the past 12 months. | sociodemographic characteristics, history of pregnancies and deliveries, mortality and morbidity of women and their children, MCH services, family planning, and attitudes towards reproductive health. | Handbook users had significantly higher odds of receiving all three kinds of medical tests and receiving information on five or more health education topics as part of antenatal care. |
| Kusumayati and Nakamura, 2007 | Indonesia | Cross-sectional | To assess the effects of utilization of the MCH handbook in Western Sumatra on the utilization of maternal health services. | MCH Handbook | Mothers who were pregnant or had children under three years of age. | Utilization of MCH handbook, utilization of MCH services. | MCHH utilization was associated with a higher likelihood of mothers utilizing ANC, TT (tetanus toxoid) immunisation, and family planning services and planned use of skilled birth attendance. |
| Mansour et al, 2019 | Lebanon | Cross-sectional (secondary data) | To assess the quality of the picture and content of the HBR itself against a pre-defined set of criteria. To compare the data found in vaccination HBRs to assess measures of vaccination status agreement between caregiver recall and HBR. | Vaccination HBR | 500 pictures of HBRs, 9315 caregivers of Lebanese and Syrian children | Picture and design quality of HBR, agreement of vaccination status between caregivers’ recall, and vaccination home-based record per vaccine dose. | Out of 9315 surveyed caregivers of Lebanese and Syrian children, 8407 (90.3%) caregivers reported that the child ever had received an HBR, yet only 5713 (61.4%) of them were able to present vaccination cards. For 3375 (36.2%) children, only caregivers’ recall was available to assess the vaccination status of the child. Only 227 children (2.4%) had never received any vaccination and did not have any available record. |
| Mudany et al, 2015 | Kenya | Cross-sectional | To assess the uptake of the mother-child health booklet by assessing the number of PCR tests done for infant HIV diagnosis and the number of MCH clinics that offered to test. | The booklet contained information on the HIV status of the mother, drugs used in pregnancy, infant and young child nutrition, immunisation records, WHO growth monitoring charts, and required actions in maternal-child emergencies. The first part of this booklet had maternal medical information during pregnancy, delivery, and the postpartum period, with the mother’s HIV status and antenatal profile. | Not reported | HIV testing in infants | Most health workers reported that the booklet made it easy for them to identify HIV-exposed infants. During the pilot period, the number of infants tested for HIV DNA increased in Nyanza from 9966 to 13 379, a 34% increase compared with a 9% overall increment in the remaining seven provinces where the booklet was not introduced. |
| Mori et al, 2015 | Mongolia | Cluster randomised trial | To assess the effectiveness of the Maternal and Child Health (MCH) handbook in Mongolia  To increase antenatal clinic attendance, and to enhance health-seeking behaviours and other health outcomes. | Maternal and Child Health handbook containing a log for recording information on maternal health and personal information, course of pregnancy, delivery and postpartum health, weight during and after pregnancy, dental health, parenting classes, child development milestones from the ages of 0–6 years, immunisation and illnesses, and height and weight charts for children. | Pregnant women and their infants living in the Bulgan province of Mongolia between May 2009 and September 2010. | Antenatal clinic attendance, health-seeking behaviours, client-provider communication, maternal physical and mental health, neonatal health, and healthy behaviour | The intervention group (253 women) attended antenatal clinics on average 6.9 times, while the control group (248 women) attended 6.2 times. Socioeconomic status affected the frequency of clinic attendance: women of higher socioeconomic status visited antenatal clinics more often. Pregnancy complications were more likely to be detected among women using the handbook. |
| Nasir et al, 2017 | Indonesia | Pre-post interventional study | To examine the effect of mother class using HBR on knowledge and practice of new-born care among mothers in the community setting. | Maternal and Child Health Handbook (MCHHB) | Mothers | Six composite outcome variables were set up: skilled birth attendance; hepatitis B immunisation, cord care, thermal protection, eye care, breastfeeding initiation. The composite variables were then categorized as a binary outcome. | Mother class has significantly improved mothers’ knowledge and practice on new-born care. Mothers might get information on new-born care during antenatal care visits at the health facilities as part of usual MCH services, but this study showed that giving knowledge about new-born care through mother class has helped the mothers to be more knowledgeable and do the good practices. Therefore, providing mother class in the community could be a method that teaches mothers how to engage in appropriate health behaviour for their babies and themselves. |
| Naidoo H et al, 2018 | South Africa | Cross-sectional | To assess the extent to which healthcare personnel, complete HIV-related, sociodemographic, neonatal, growth, and immunisation information in the RTHC and/or RTHB. | Road-to-Health Booklet (RTHB), or the older, less detailed, Road-to- Health Card/Chart (RTHC). | Children aged less than two years attending the paediatric departments | HIV testing, sociodemographic and neonatal completeness | 24% of all RTHBs had no record of maternal HIV status and 67% of RTHBs from documented HIV-exposed infants had no record of maternal ART duration.  Neonatal information completeness was similar between RTHBs and RTHCs. |
| Osaki et al, 2019 | Indonesia | Cluster randomised trial | To assess the effect of MCH handbook use in rural  Java, where service coverage was comparatively low. | Maternal and Child Health Handbook | Pregnant women attending the health centres. | Maternal immunisation, antenatal clinic appointments, vitamin A intake, feeding practices, child growth, and development. | Respondents in the intervention area received consecutive MCH services including two doses of tetanus toxoid injections and antenatal care four times or more during pregnancy, professional assistance during child delivery, and vitamin A supplements administration to their children, after adjustment for confounding variables and cluster effects (OR =2.03, 95% CI: 1.19–3.47). In the intervention area, home care (continued breastfeeding; introducing complementary feeding; proper feeding order; varied foods feeding; self-feeding training; and care for cough), perceived support by husbands, and lower underweight rates and stunting rates among children were observed |
| Osaki et al, 2013 | Indonesia | Cross-sectional | To identify the roles of home-based records both before and after childbirth, especially in provinces where the MCH handbook (MCHHB) was extensively promoted, by examining their association with MNCH service uptake | MCH handbook | Using nationally representative data sets, the Indonesia Demographic and Health Surveys (IDHSs) from 1997, 2002, 2003, and 2007. The IDHS identifies respondents’ ownership of home-based records before and after childbirth. |  | Provincial data from 2007 showed that handbook ownership was associated with having delivery assisted by trained personnel [adjusted odds ratio (aOR): 2.12, 95% confidence interval (CI): 1.054.25], receiving maternal care (aOR: 3.92, 95% CI: 2.356.52), completing 12 doses of child immunisation for seven diseases (aOR: 4.86, 95% CI: 2.379.95), and having immunisation before and after childbirth (aOR: 5.40, 95% CI: 2.2812.76), whereas national data showed that service utilisation was associated with ownership of both records compared with owning a single record or none. |
| Ramraj T et al, 2018 | South Africa | Cross-sectional | To assess the completeness of patient-held infant Road to Health Booklets (RTHBs), amongst HIV exposed and unexposed infants during the first two years after the RTHB was launched country-wide in South Africa | Road-to-Health Booklet (RTHB), | Infants aged 4-8 weeks who were receiving their six-week immunisation on the day of visit and who did not need emergency care | Completeness of the RTHB - that should have been completed at birth, namely; infant birth weight, BCG immunisation, maternal HIV status, and an indication of whether maternal syphilis testing was done. | Overall, recording of all four indicators increased from 23.1% (95% confidence interval (CI) = 22.2-24.0) in 2011-12 to 43.3% (95% CI = 42.3-44.4) in 2012-13. |
| Silva et al, 2015 | Brazil | Qualitative study | To analyse the use of child health records by families, based on the perceptions of health professionals, in pursuit of new support for integrated health care for children. | Child Health Record | Family and mothers | The use of child health records by families. | The study showed that HCPs believe health records are an instrument of communication and education for families. There is a concern about instructing mothers and families about the importance of keeping track of their children’s health, in addition to the purpose, content and relevance of health records. Professionals also believed that, despite the guidance provided, families seldom use this instrument. The study participants also recognized the right of families to demand the recording of data in records; this attitude showed that families are interested in their children’s health, and also assists in the work of professionals. |
| Shah et al, 1993 | (Egypt, India, Pakistan, Philippines, Senegal,  Sri Lanka, Yemen and Zambia) | Pre-post surveys | To evaluate the function of the HBMR (a home-based maternal record) following a set of WHO guidelines. | Home-based maternal record (HBMR) | Mothers and a range of HCPs including community health workers, skilled birth attendants, nurses, and physicians | Use of HBMR, Identification of risk conditions, and referral care | Substantial improvement in maternal and neonatal care, and continuity of care in areas using HBMR. (Examples: Philippines 91–100% vs. 36.6–51.9%; Zambia 93.5% vs. 49.8%). Records adapted to the local situation. Improvement was noted in maternal knowledge for self-care. |
| Palombo et al, 2014 | Brazil | Cross-sectional | To evaluate the use and records of the Child Health Handbook (CHH), especially | Child Health Handbook | Mother and child pairs | The use of the CHH was evaluated with the following questions: Did the mother have the CHH at the time of the interview? | Fifty-one percent of the mothers were carrying the CHH at the time of the interview, similar to the proportion of mothers who were instructed to bring the CHH to health appointments. Annotations in the CHH during the visits were reported by 49%. The vaccination schedule was completed in 97% of the CHH, but only 9% and 8% of the CHH, respectively, contained growth charts and properly completed developmental milestones. |
| Tarwa et al, 2007 | South Africa | Quantitative descriptive study | To assess whether the Road to Health Card (RTHC)s are completed and interpreted adequately at primary, secondary, and tertiary care levels in South Africa. | Road to Health Card (RTHC) | Caregivers who accompanied children younger than five years of age. | Information on whether the RTHC had been brought along and, reasons for not bringing. | The RTHC was not brought to 48% of the consultations. Of these respondents, about 72% thought that bringing along the RTHC was not necessary. Health workers seldom asked to see the RTHC in the primary and secondary care settings, but 50% of them did so at Hospital (p = 0,002). |
| Tjandraprawira et al, 2018 | Indonesia | Cross-sectional study | To investigate its role in increasing awareness of Indonesian women about the various obstetric  danger signs and the entailing complication. | Mother and Child Health (MCH) handbook | Recently delivered postpartum women treated in the maternity ward of Majalengka General District Hospital. | The average score among ≥ 50% MCHH readers Average score among < 50% MCHH readers Average questionnaire score | The MCH handbook did not hold a significant role in effecting this finding (p-value 0.295). No significant influence in improving maternal knowledge levels around pregnancy and the associated obstetric danger signs. |
| Gonzalez et al, 2019 | Brazil | Cross-sectional study | To measure the prevalence of acquiring and evaluating the level of completion of the pregnant women’s medical booklet on the occasion of childbirth in Rio Grande, Brazil | Pregnant women’s medical booklet | All the parturient living in the urban and rural areas in Rio Grande | The prevalence of the outcome and the frequency of completion of the information registered in the pregnant woman’s medical booklet among the surveys were compared | Out of 10,242 pregnant women in this study 54.8% (95% CI 53.8%-55.7%) had their pregnant woman’s medical booklet with them at the time of admission. The completion pattern of the pregnant woman’s medical booklet is divided into three groups, namely: with at least 95%: date of the last consultation visit, maternal height and blood pressure verification, uterine height, cardio-fetal heart rate, and the Rh factor; 85% or more: date of the last menstruation, qualitative urine test, VDRL (Venereal disease research laboratory test) and HIV; and less than 30%: performance of clinical breast examination and cytopathology of the uterine cervix. The use of the pregnant woman’s medical booklet and its completion were lower than expected on several items. |
| Usman et al, 2009 | Pakistan | RCT | To assess the effect of a redesigned immunisation card and centre-based education on mothers on DTP3 completion. | Redesigned immunisation card | Children visiting the selected EPI centres for DTP1 and residing in the same area for the last 6 months. | Immunisation status of 3-dose DTP vaccination. | A significant increase of 31% (adjusted RR = 1.31, 95% CI = 1.18–1.46) in DTP3 completion was estimated in the group that received both redesigned cards and centre-based education compared with the standard care group. |
| Usman et al, 2011 | Pakistan | RCT | To assess the effects of providing substantially redesigned immunisation cards, centre-based education, or both interventions together on DTP3 completion at six rural Expanded Programme on Immunisation (EPI) centres in Pakistan. | Redesigned immunisation card | Children visiting the selected EPI centres for DTP1 and residing in the same area for the last 6 months. | The immunisation status of 3-dose DTP vaccination. | 39% of children in standard care group completed DTP3. Compared to this, a significantly higher proportion of children completed DTP3 in redesigned card group (66%) (Crude Risk Ratio [RR] = 1.7; 95% CI = 1.5, 2.0), centre-based education group (61%) (RR = 1.5; 95% CI = 1.3, 1.8), and combined intervention group (67%) (RR = 1.7; 95% CI = 1.4, 2.0). |
| Vieira et al, 2017 | Brazil | Cross-sectional study | To investigate the factors associated with mothers reading the Child Health Handbook (CHH) and health professionals completing this instrument, in Feira de Santana, Bahia, 2009. | Child Health Handbook | Children under one year of age up to the day of the survey, from the municipality of Feira de Santana, who attended the vaccination units accompanied by their respective mothers and/or caregiver. | Mother reading the CHH and health professionals completing weight and height measures. | The prevalence of reading, weight, and height was, respectively, 81.1%, 68.9%, and 47.3%. Mothers with a higher level of education had a greater chance of reading the CHH. |
| Wallace et al, 2019 | Indonesia | Cluster RCT | To estimate the effect of low-cost parental reminder interventions using HBRs on completion and timeliness of the 3-dose DTP cv series. | Child's HBR (vaccination reminder) | All children who received DTPcv1 in a study health facility in January 2016 and had the vaccination recorded on the facility vaccination register | The primary outcome was the receipt of the third dose of diphtheria-tetanus-pertussis-containing vaccine (DTPcv3) within 7 months and the secondary outcome was the receipt of a timely DTPcv3 dose. | In intention-to-treat analysis, neither intervention group had significantly different DTPcv3 coverage compared with the control group (RR = 0.94, 95% confidence interval [CI] 0.87; 1.02 for HBR-only group; RR = 0.97, 95% CI 0.90; 1.04 for HBR + sticker group) by study end. However, children in the HBR + sticker group were 50% more likely to have received a DTPcv3 vaccination (RR = 1.46, 95% CI 1.02, 2.09) within 60 days of DTPcv1 vaccination, compared with children in the control group. |
| Yanagisawa et al, 2015 | Cambodia | Pre-post study | To assess the effectiveness of the MCH handbook in Cambodia | MCH Handbook | Women who had given birth 1 year before the survey lived in an intervention or control area. | Maternal behaviours include antenatal attendance, deliveries attended by SBAs, and deliveries at health facilities). Secondary outcomes included maternal knowledge of danger signs during pregnancy and delivery, prevention of anaemia, prevention of intestinal parasites, mother-to-child HIV transmission, early breastfeeding practice and child immunisation | The intervention increased ANC attendance, delivery with SBAs and delivery at a health facility, even after adjusting for maternal age, education and economic conditions. |

HBR=home-based records, MCH=maternal and child health, SBA=skilled birth attendant, ANC=antenatal care, cRCT=cluster randomised controlled trial, HIV=Human Immunodeficiency virus,

Table S3: Results of Mixed Methods Appraisal Tool

| Author, year | RANDOMIZED CONTROLLED TRIALS | | | |  |  |
| --- | --- | --- | --- | --- | --- | --- |
|  | Is randomization appropriately performed? | Are the groups comparable at baseline? | Are there complete outcome data? | Are outcome assessors blinded to the intervention provided? | Did the participants adhere to the assigned intervention? | Score |
| Usman, 2009 | Yes | Yes | Yes | No | Yes | 4 |
| Usman,2011 | Yes | Yes | Yes | No | Yes | 4 |
| Dagvadorj, 2017 | Yes | Yes | No | No | Yes | 3 |
| Mori, 2015 | Yes | Yes | Yes | No | Yes | 4 |
| Osaki ,2019 | Yes | Yes | No | No | Yes | 3 |
| Wallace, 2019 | Can’t tell | Yes | Yes | No | Yes | 3 |
|  | NON-RANDOMIZED STUDIES | | | | |  |
|  | Are the participants representative of the target population? | Are measurements appropriate regarding both the outcome and intervention (or exposure)? | Are there complete outcome data? | Are the confounders accounted for in the design and analysis? | During the study period, is the intervention administered (or exposure occurred) as intended? |  |
| Aiga et al, 2016 | Yes | Yes | Yes | No | Yes | 4 |
| Hagiwara et al, 2008 | No | Yes | Yes | Yes | Yes | 4 |
| Kaneko et al, 2017 | Can't tell | Yes | Yes | Yes | Yes | 4 |
| Shah et al, 1998 | No | Yes | No | No | Can't tell | 1 |
| Yanagisawa et al, 2015 | Yes | Yes | Yes | Yes | Yes | 5 |
| Bhuiyan et al, 2006 | Can't tell (no sampling frame description) | Yes | Yes | No | Yes | 3 |
| Nasir et al, 2017 | Yes | Yes | Yes | Yes | Can’t tell | 4 |
| Harrison et al, 1998 | Can't tell | Yes | Yes | No | Yes | 3 |
|  | QUANTITATIVE DESCRIPTIVE STUDIES | | | | |  |
|  | Is the sampling strategy relevant to address the research question? | Is the sample representative of the target population? | Are the measurements appropriate? | Is the risk of nonresponse bias low? | Is the statistical analysis appropriate to answer the research question? |  |
| Kusumayati and Nakamura, 2007 | Can't tell | Can't tell | Yes | Can't tell | Yes | 2 |
| Tarwa et al, 2007 | Can't tell | No | Yes | Can't tell | Yes | 2 |
| Mudany et al, 2015 | No | Can't tell | Can't tell | Can't tell | Yes | 1 |
| Jahn et al, 2008 | Yes | Yes | Yes | Yes | Yes | 5 |
| Hayford et al 2013 | Yes | Yes | Yes | No | Yes | 4 |
| Vieira et al,2017 | Yes | No | Yes | Can't tell | Yes | 3 |
| Hikita et al, 2018 | Yes | Yes | Yes | Yes | Yes | 5 |
| Palombo et al, 2014 | Yes | Yes | Yes | No | Yes | 4 |
| Kitabayashi et al, 2017 | Yes | Yes | Yes | Can't tell | Yes | 4 |
| Osaki et al, 2013 | Yes | Yes | Yes | Can't tell | Yes | 4 |
| Kawakatsu et al, 2015 | Yes | Yes | Yes | Can't tell | Yes | 4 |
| Baequni et al, 2016 | Yes | Can’t tell | Yes | Can’t tell | Yes | 3 |
| Brown et al, 2018 | Yes | Yes | Yes | Can't tell | Yes | 4 |
| Ramraj et al, 2018 | Yes | Yes | Yes | Can't tell | Yes | 4 |
| Naidoo et al,2018 | No | Yes | Yes | Can't tell | Yes | 3 |
| Tjandraprawira et al, 2018 | No | No | Yes | Can't tell | Yes | 2 |
| Kabore et al, 2020 | Yes | No | Yes | Yes | Yes | 4 |
| Abud and Gaiva, 2015 | Yes | Yes | Yes | Can’t tell | Yes | 4 |
| Camargos et al, 2021 | Yes | No | Yes | Can’t tell | Yes | 3 |
| Coehlo et al, 2021 | Yes | No | Yes | Can’t tell | Yes | 3 |
| Araujo et al, 2017 | Yes | No | Yes | Yes | Yes | 4 |
| Amorim et al, 2018 | Yes | Yes | Yes | Can’t tell | Yes | 4 |
| Gonzalez et al, 2019 | Yes | Yes | Yes | Can’t tell | Yes | 4 |
| Adedire et al, 2016 | Yes | Yes | Yes | Yes | Yes | 5 |
| Mansour et al, 2019 | Yes | Yes (Only one district out of 26 excluded with proper justification) | Yes | Yes | Yes | 5 |
|  | QUALITATIVE STUDIES | | | | | |
|  | Is the qualitative approach appropriate to answer the research question? | Are the qualitative data collection methods adequate to address the research question? | Are the findings adequately derived from the data? | Is the interpretation of results sufficiently substantiated by data? | Is there coherence between qualitative data sources, collection, analysis and interpretation? |  |
| Andrade et al, 2014 | Yes | Yes (exploratory, descriptive) | Yes | Can't tell | Can't tell | 3 |
| Silva et al, 2015 | Yes | Yes | Yes | Yes | Yes | 5 |
|  | MIXED METHODS STUDIES | | | | |  |
|  | Is there an adequate rationale for using a mixed methods design to address the research question? | Are the different components of the study effectively integrated to answer the research question? | Are the outputs of the integration of qualitative and quantitative components adequately interpreted? | Are divergences and inconsistencies between quantitative and qualitative results adequately addressed? | Do the different components of the study adhere to the quality criteria of each tradition of the methods involved? |  |
| Gustaffson et al, 2020 | Yes | Yes | Can't tell (Not clear from the paper) | No | Yes | 3 |

S4: Systematic review protocol registered in PROSPERO (CRD42019139365)

[P](http://www.nihr.ac.uk/)atient-held records in low- and middle-income countries (LMICs): a systematic review

Review question

To explore the literature around patient-held records in low- and middle-income countries.

In particular, we wish to answer the following question:

Can patient-held record use in LMICs improve clinical handover, patient-centred care, and self-care management?

In addition, we wish to:

1. Summarize the evidence on patient-held records in improving clinical handover, patient-centred care, and self-care management.
2. Investigate user perceptions of patient-held record use.

Searches

The search strategy will be developed for electronic databases such as MEDLINE (Ovid MEDLINE(R), Ovid MEDLINE(R) In-Process & Other Non-Indexed Citations, Ovid MEDLINE(R) Daily and Ovid OLDMEDLINE(R) (1946 to present)), EMBASE (Ovid EMBASE Classic + EMBASE (1947 to present)); and CINAHL (via EBSCO (1980 to present)).

Also, a lateral search using any relevant systematic reviews to locate additional relevant studies will be carried out.

In addition, the grey literature search will also be searched to identify documents relating to available guidance on patient-held records (OpenGrey).

Key search terms will be related to “patient held records” and “low-middle income countries”.

The searches in the electronic databases will be carried out from their respective inception dates to November 2018.

No limits will be imposed on language or publication year during the searches, although studies published in languages other than English will not be included.

Additional search strategy information can be found in the attached PDF document (link provided below).

Types of study to be included

All study designs will be included: intervention studies with patient-held records vs no records or usual care, non-intervention studies including information on handover communication, and qualitative studies on the perceptions of users.

Inclusion criteria:

1. Any patient-held health information, which provides a continued record of care, which has been designed for use by both healthcare providers and patients. 2. Studies including paper-based records (which will be classed as hand-held). If electronic, the records will be accessible by hospital clinicians, patients, and community clinicians.

3. Studies that have reported the use of patient-held records for the transfer or exchange of information across care transitions.

Exclusion criteria:

1. Any paper or electronic record that is a hospital-based medical chart, or facility-based medical record.
2. Studies, which have reported on the tools specific to one or more transitions of, care (i.e. hospital shift-change, referral, transfer, or discharge) such as discharge summaries or referral letters. 3. Patient diaries and mobile health interventions that involve text messages will not be considered to be eligible interventions.

Condition or domain being studied

Patient-held records in low- and middle-income countries. All disease conditions will be included, provided the associated intervention involves the use of patient-held records.

Participants/population

Participants (patients, carers, and healthcare workers) of any age and sex from low- and middle-income countries (as defined by the World Bank in 2018).

Intervention(s), exposure(s)

Any intervention that uses patient-held records. Examples of patient-held records include: maternal and child health cards, diabetes passports, chronic disease booklets, person-specific patient-held handbooks, etc.

For the purposes of this systematic review, patient-held records are defined as continued care records (with health information recorded by health care providers) handed over to the custody of the person, which contributes to handover communication and continuity of care.

Comparator(s)/control

None.

Context

Main outcome(s)

Patient-held records in low- and middle-income countries (LMICs).

Measurements and reports of the process or clinical outcomes will be considered, as follows:

Process outcomes relating to handovers (the record being a continued record) such as the number of completed entries in the patient-held record, or appointment-keeping, legibility, or accuracy of the information recorded.

Relevant clinical outcomes highlight an improvement in patient care continuity.

Patient- and health care provider-reported outcomes of satisfaction with the care or the care continuity.

Additional outcomes will include factors (e.g. barriers, facilitators, etc.) that are reported as affecting the use of patient-held records.

*Measures of effect* Not applicable.

Additional outcome(s)

Additional information on the development of the patient-held record, if reported.

*Measures of effect* No time limitations.

Data extraction (selection and coding)

Two reviewers (LJ and DB) from the studies retrieved during the searches will select eligible studies.

The initial title and abstract selection will be broad, with studies mentioning patient-held records being retained, to facilitate further reference searching for additional papers.

Following this, the full texts of the retained literature will be sought, and those that can be accessed will be screened against the aforementioned inclusion criteria.

If there is a degree of uncertainty surrounding the inclusion of a study or any disagreement following discussions, an additional reviewer (JP) will be available to provide a conclusive assessment.

Data extraction will be split between available members of the research team (DB, PJ, LAJ), with LJ randomly sampling 10% of all studies to ensure reliability in the process. If there are any discrepancies during this sampling check, discussions will take place, and if a resolution cannot be reached, then a third impartial reviewer (JP or SMH) will be called upon for a final decision. LJ will be responsible for organising the data and will check for data entry discrepancies.

An electronic data extraction form has been drafted in Excel, piloted, and is ready for final use.

The data to be extracted will include details of the studies (authors, settings, year), the features of the patient-held records, the aims and objectives of the studies, study designs, the information recorded and communicated by the studies, the outcome measures relating to handover, patient-centred care, self-management, the clinical outcomes, the user perceptions of the patient-held records, and the findings from the studies.

Risk of bias (quality) assessment

The quality assessment will be carried out by two reviewers (LJ and DB). Given the mixed-methods nature of this review, the Mixed Method Appraisal Tool (MMAT) will be used, which is a reliable instrument that has been used for several mixed-methods systematic reviews covering literature across the high-, middle- and low-income countries.

Strategy for data synthesis

The review will be summarised descriptively. A meta-analysis will not be carried out as a large degree of heterogeneity is expected in the different types of patient-held records for different diseases, and the different tools for evaluation.

First, a preliminary synthesis of the included studies will be undertaken by listing and presenting the results in tabular form.

The analysis will look into how studies have used patient-held records for handover and will be structured using the outcome measures and user perspectives. Following this, results will be mapped onto a framework, as explained by Osaki et al, for the functions and use of home-based records. This will enable in better understanding of the function of the records (for handover), and the extent to which the users of the records (patients and health care providers) have the knowledge and skill to carry out that function, as expected.

Analysis of subgroups or subsets

None planned.

S5: Example search strategy in EMBASE

Patient-held records

1. (exp medical records/or exp medical records, problem-oriented/or exp medical records systems, computerized/or exp nursing records/or (((medical or health) and record*).mp. or (case note* or case record*).mp.) or (record* or book* or handbook* or card*).mp AND ((held adj3 (patient or parent or person or woman or man or family or consumer)).mp. or (carried adj3 (patient or parent or person or woman or man or family or client or consumer)).mp.)) OR ((log-book* or logbook*).mp. or home based record*.mp. or passport*.mp. or personal health record*.mp.)
2. ((patient-held or patient-held or home based or home-based or personal child or "mother and child" or "maternal and child" or mother-child) adj2 (record* or book* or handbook* or card*)).mp
3. (( patient access or personal or patient-held) adj2( record*or portal * or health record* or information* or prescription* or electronic record*)).mp
4. 1 or 2 or 3

Low-and Middle-income countries

1. exp developing country/ or exp medically underserved/ or developing countr$.mp. or medically underserved area$1.mp. or low income countr$.mp. or middle income country.mp. or low resource.mp. or resource poor.mp. or global.mp. or exp Africa/ or exp "South and Central America"/or exp asia/ or exp Caribbean islands/ or exp pacific islands/ or exp eastern Europe/ or exp Indian Ocean/or south america$1.mp. or Africa$1.mp. or Caribbean.mp. or central America$1.mp. or south America$1.mp. or eastern Europe$1.mp. or pacific island$.mp. or Indian ocean island$.mp. or asia.mp. or Afghan$.mp. or Bangladesh$1.mp. or Benin$.mp. or Burkina Faso.mp. or Burkinabe.mp. or Burundi$.mp. or Cambodia$1.mp. or Central African.mp. or Chad$.mp. or Comor$.mp. or Congo$.mp. or Eritrea$1.mp. or Ethiopia$1.mp. or Gambia$1.mp. or Guinea$1.mp. or Haiti$.mp. or Kenya$1.mp. or Korea$1.mp. or exp North Korea/ or Kyrgyz$.mp. or Liberia$1.mp. or Madagascar.mp. or Malagasy.mp. or Malawi$.mp. or mali$.mp. or mozambi$.mp. or Myanmar$.mp. or Nepal$.mp. or Niger$.mp. or Rwanda$1.mp. or Sierra Leone$.mp. or Somalia$1.mp. or Tajik$.mp. or Tanzania$1.mp. or Togo$.mp. or Uganda$1.mp. or Zimbabwe$.mp. or Angola$1.mp. or Armenia$1.mp. or Beliz$.mp. or Bhutan$.mp. or Bolivia$1.mp. or Cameroon$.mp. or Cape Verde$.mp. or Congo$.mp. or "Côte d'Ivoire".mp. or Ivory Coast.mp. or Ivorian.mp. or Djibouti.mp. or Egypt$.mp. or El Salvador.mp. or Salvadoran.mp. or Fiji$.mp. or Georgia$1.mp. or Ghana$.mp. or Guatemala$1.mp. or Guyan$.mp. or Hondura$.mp. or Indonesia$1.mp. or India$1.mp. or Iraq$1.mp. or Kiribati.mp. or Kosov$.mp. or Lao$.mp. or Lesotho.mp. or Marshall Islands.mp. or Marshallese.mp. or Mauritania$1.mp. or Micronesia$1.mp. or Moldov$.mp. or Mongolia$1.mp. or Morocc$.mp. or Nicaragua$1.mp. or Nigeria$1.mp. or Pakistan$1.mp. or Papua New Guinea$1.mp. or Paraguay$.mp. or Philippines.mp. or Filipino.mp. or Samoa$1.mp. or sao tome$.mp. or Senegal$.mp. or Solomon Island$.mp. or sri lanka$1.mp. or Sudan$.mp. or Swazi$.mp. or Syria$1.mp. or Timor$.mp. or Tonga$1.mp. or Turkmen$.mp. or Tuvalu$.mp. or Ukrain$.mp. or Uzbek$.mp. or Vanuat$1.mp. or Vietnam$.mp. or West Bank.mp. or Gaza.mp. or Yemen$.mp. or Zambia$1.mp. or Albania$1.mp. or Algeria$1.mp. or "Antigua and Barbuda".mp. or antiguan.mp. or barbudan.mp. or Azerbaijan$1.mp. or Belarus$.mp. or Bosnia$1.mp. or Botswana.mp. or Brazil$.mp. or Bulgaria$1.mp. or Chile$.mp. or China.mp. or Chinese.mp. or Colombia$1.mp. or Costa Rica$1.mp. or Cuba$1.mp. or Dominica$1.mp. or Ecuador$.mp. or Gabon$.mp. or Grenad$.mp. or Iran$.mp. or Jamaica$1.mp. or Jordan$.mp. or Kazakhstan$1.mp. or Latvia$1.mp. or Leban$.mp. or Libya$1.mp. or Lithuania$1.mp. or Macedonia$1.mp. or Malaysia$1.mp. or Maldiv$.mp. or mauriti$.mp. or Mexic$.mp. or Montenegr$.mp. or Namibia$1.mp. or Palau$.mp. or Panama$.mp. or Peru$.mp. or Romania$1.mp. or Russia$1.mp. or Serbia$1.mp. or Seychell$.mp. or South Africa$1.mp. or Saint Kitts.mp. or Saint Lucia.mp. or Saint Vincent.mp. or Suriname$1.mp. or Thai$.mp. or Tunisia$1.mp. or Turk$.mp. or Uruguay$.mp. or Venezuela$1.mp
2. 4 and 5

S6: PRISMA Checklist

| Section/topic | # | Checklist item | Reported on page # |
| --- | --- | --- | --- |
| TITLE | | |  |
| Title | 1 | Identify the report as a systematic review, meta-analysis, or both. | 1, Title page |
| ABSTRACT | | |  |
| Structured summary | 2 | Provide a structured summary including, as applicable: background; objectives; data sources; study eligibility criteria, participants, and interventions; study appraisal and synthesis methods; results; limitations; conclusions and implications of key findings; systematic review registration number. | 2, Abstract |
| INTRODUCTION | | |  |
| Rationale | 3 | Describe the rationale for the review in the context of what is already known. | 5 |
| Objectives | 4 | Provide an explicit statement of questions being addressed with reference to participants, interventions, comparisons, outcomes, and study design (PICOS). | 5,7 |
| METHODS | | |  |
| Protocol and registration | 5 | Indicate if a review protocol exists, if and where it can be accessed (e.g., Web address), and, if available, provide registration information including registration number. | 6 |
| Eligibility criteria | 6 | Specify study characteristics (e.g., PICOS, length of follow-up) and report characteristics (e.g., years considered, language, publication status) used as criteria for eligibility, giving rationale. | 7,8 |
| Information sources | 7 | Describe all information sources (e.g., databases with dates of coverage, contact with study authors to identify additional studies) in the search and date last searched. | 9 |
| Search | 8 | Present full electronic search strategy for at least one database, including any limits used, such that it could be repeated. | Supplementary file, S5 |
| Study selection | 9 | State the process for selecting studies (i.e., screening, eligibility, included in systematic review, and, if applicable, included in the meta-analysis). | 9 |
| Data collection process | 10 | Describe method of data extraction from reports (e.g., piloted forms, independently, in duplicate) and any processes for obtaining and confirming data from investigators. | 9 |
| Data items | 11 | List and define all variables for which data were sought (e.g., PICOS, funding sources) and any assumptions and simplifications made. | 9 |
| Risk of bias in individual studies | 12 | Describe methods used for assessing risk of bias of individual studies (including specification of whether this was done at the study or outcome level), and how this information is to be used in any data synthesis. | 10 |
| Summary measures | 13 | State the principal summary measures (e.g., risk ratio, difference in means). | NA |
| Synthesis of results | 14 | Describe the methods of handling data and combining results of studies, if done, including measures of consistency (e.g., I^2^) for each meta-analysis. | 10,11 |

Box S2: Search strategy in Ovid MEDLINE(R) In-Process & Other Non-Indexed Citations, Ovid MEDLINE(R) Daily and Ovid MEDLINE(R) <1946 to Present>

| Patient-held records   1. (exp medical records/or exp medical records, problem-oriented/or exp medical records systems, computerized/or exp nursing records/or (((medical or health) and record*).mp. or (case note* or case record*).mp.) or (record* or book* or handbook* or card*).mp AND ((held adj3 (patient or parent or person or woman or man or family or consumer)).mp. or (carried adj3 (patient or parent or person or woman or man or family or client or consumer)).mp.)) OR ((log-book* or logbook*).mp. or home based record*.mp. or passport*.mp. or personal health record*.mp.) 2. ((patient-held or patient-held or home based or home-based or personal child or "mother and child" or "maternal and child" or mother-child) adj2 (record* or book* or handbook* or card*)).mp 3. (( patient access or personal or patient-held) adj2( record*or portal * or health record* or information* or prescription* or electronic record*)).mp 4. 1 or 2 or 3   Low-and Middle-income countries   1. exp developing country/ or exp medically underserved/ or developing countr$.mp. or medically underserved area$1.mp. or low income countr$.mp. or middle income country.mp. or low resource.mp. or resource poor.mp. or global.mp. or exp Africa/ or exp "South and Central America"/or exp asia/ or exp Caribbean islands/ or exp pacific islands/ or exp eastern Europe/ or exp Indian Ocean/or south america$1.mp. or Africa$1.mp. or Caribbean.mp. or central America$1.mp. or south America$1.mp. or eastern Europe$1.mp. or pacific island$.mp. or Indian ocean island$.mp. or asia.mp. or Afghan$.mp. or Bangladesh$1.mp. or Benin$.mp. or Burkina Faso.mp. or Burkinabe.mp. or Burundi$.mp. or Cambodia$1.mp. or Central African.mp. or Chad$.mp. or Comor$.mp. or Congo$.mp. or Eritrea$1.mp. or Ethiopia$1.mp. or Gambia$1.mp. or Guinea$1.mp. or Haiti$.mp. or Kenya$1.mp. or Korea$1.mp. or exp North Korea/ or Kyrgyz$.mp. or Liberia$1.mp. or Madagascar.mp. or Malagasy.mp. or Malawi$.mp. or mali$.mp. or mozambi$.mp. or Myanmar$.mp. or Nepal$.mp. or Niger$.mp. or Rwanda$1.mp. or Sierra Leone$.mp. or Somalia$1.mp. or Tajik$.mp. or Tanzania$1.mp. or Togo$.mp. or Uganda$1.mp. or Zimbabwe$.mp. or Angola$1.mp. or Armenia$1.mp. or Beliz$.mp. or Bhutan$.mp. or Bolivia$1.mp. or Cameroon$.mp. or Cape Verde$.mp. or Congo$.mp. or "Côte d'Ivoire".mp. or Ivory Coast.mp. or Ivorian.mp. or Djibouti.mp. or Egypt$.mp. or El Salvador.mp. or Salvadoran.mp. or Fiji$.mp. or Georgia$1.mp. or Ghana$.mp. or Guatemala$1.mp. or Guyan$.mp. or Hondura$.mp. or Indonesia$1.mp. or India$1.mp. or Iraq$1.mp. or Kiribati.mp. or Kosov$.mp. or Lao$.mp. or Lesotho.mp. or Marshall Islands.mp. or Marshallese.mp. or Mauritania$1.mp. or Micronesia$1.mp. or Moldov$.mp. or Mongolia$1.mp. or Morocc$.mp. or Nicaragua$1.mp. or Nigeria$1.mp. or Pakistan$1.mp. or Papua New Guinea$1.mp. or Paraguay$.mp. or Philippines.mp. or Filipino.mp. or Samoa$1.mp. or sao tome$.mp. or Senegal$.mp. or Solomon Island$.mp. or sri lanka$1.mp. or Sudan$.mp. or Swazi$.mp. or Syria$1.mp. or Timor$.mp. or Tonga$1.mp. or Turkmen$.mp. or Tuvalu$.mp. or Ukrain$.mp. or Uzbek$.mp. or Vanuat$1.mp. or Vietnam$.mp. or West Bank.mp. or Gaza.mp. or Yemen$.mp. or Zambia$1.mp. or Albania$1.mp. or Algeria$1.mp. or "Antigua and Barbuda".mp. or antiguan.mp. or barbudan.mp. or Azerbaijan$1.mp. or Belarus$.mp. or Bosnia$1.mp. or Botswana.mp. or Brazil$.mp. or Bulgaria$1.mp. or Chile$.mp. or China.mp. or Chinese.mp. or Colombia$1.mp. or Costa Rica$1.mp. or Cuba$1.mp. or Dominica$1.mp. or Ecuador$.mp. or Gabon$.mp. or Grenad$.mp. or Iran$.mp. or Jamaica$1.mp. or Jordan$.mp. or Kazakhstan$1.mp. or Latvia$1.mp. or Leban$.mp. or Libya$1.mp. or Lithuania$1.mp. or Macedonia$1.mp. or Malaysia$1.mp. or Maldiv$.mp. or mauriti$.mp. or Mexic$.mp. or Montenegr$.mp. or Namibia$1.mp. or Palau$.mp. or Panama$.mp. or Peru$.mp. or Romania$1.mp. or Russia$1.mp. or Serbia$1.mp. or Seychell$.mp. or South Africa$1.mp. or Saint Kitts.mp. or Saint Lucia.mp. or Saint Vincent.mp. or Suriname$1.mp. or Thai$.mp. or Tunisia$1.mp. or Turk$.mp. or Uruguay$.mp. or Venezuela$1.mp 2. 4 and 5 |
| --- |

1. Osaki K, Aiga H. Adapting home-based records for maternal and child health to users' capacities. Bull World Health Organ. 2019 Apr 1;97(4):296-305. doi: 10.2471/BLT.18.216119. Epub 2019 Feb 14. PMID: 30940987; PMCID: PMC6438250. [↑](#footnote-ref-1)
2. Brown DW, Bosch-Capblanch X, Shimp L. Where Do We Go From Here? Defining an Agenda for Home-Based Records Research and Action Considering the 2018 WHO Guidelines. Glob Health Sci Pract. 2019 Mar 29;7(1):6-11. doi: 10.9745/GHSP-D-18-00431. PMID: 30877139; PMCID: PMC6538131. [↑](#footnote-ref-2)
